# Gender Is the Main Predictor of Wearing‐Off and Dyskinesia in Levodopa‐Naïve Patients with Parkinson's Disease

**DOI:** 10.1002/mdc3.70143

**Published:** 2025-05-29

**Authors:** Maria Teresa Pellecchia, Marina Picillo, Maria Claudia Russillo, Marianna Amboni, Gennarina Arabia, Laura Avanzino, Margherita Canesi, Alessia Catania, Roberto Ceravolo, Calogero Edoardo Cicero, Roberto Cilia, Isabel Colangelo, Giovanna Dati, Rosa De Micco, Anna De Rosa, Alessio Di Fonzo, Roberto Eleopra, Vincenza Fetoni, Barbara Garavaglia, Augusta Giglio, Martina Giuntini, Federica Invernizzi, Giulia Lazzeri, Roberta Marchese, Alessandra Nicoletti, Claudio Pacchetti, Celeste Panteghini, Manuela Pilleri, Fabiana Radicati, Chiara Reale, Cesa Scaglione, Andrea Soricelli, Fabrizio Stocchi, Alessandro Tessitore, Laura Vacca, Maria Antonietta Volontè, Graziella Volpi, Roberta Zangaglia, Francesco Amato, Carlo Ricciardi, Michela Russo, Paolo Barone

**Affiliations:** ^1^ Department of Medicine, Surgery and Dentistry “Scuola Medica Salernitana,” Neuroscience Section University of Salerno Salerno Italy; ^2^ Department of Medical and Surgical Sciences, Institute of Neurology Magna Græcia University Catanzaro Italy; ^3^ Department of Experimental Medicine (DIMES), Section of Human Physiology University of Genoa Genoa Italy; ^4^ Neurorehabilitation Unit, Parkinson's Disease and Movement Disorders Center Moriggia‐Pelascini Hospital Como Italy; ^5^ Medical Genetics and Neurogenetics Unit Fondazione IRCCS Istituto Neurologico “C. Besta” Milano Italy; ^6^ Department of Clinical and Experimental Medicine University of Pisa Pisa Italy; ^7^ Neurologic Unit, AOU “Policlinico‐San Marco,” Department of Medical, Surgical Sciences and Advanced Technologies, GF Ingrassia University of Catania Catania Italy; ^8^ Department of Clinical Neurosciences, Parkinson and Movement Disorders Unit Fondazione IRCCS Istituto Neurologico Carlo Besta Milan Italy; ^9^ Department of Advanced Medical and Surgical Sciences University of Campania “Luigi Vanvitelli” Naples Italy; ^10^ Department of Neurosciences and Reproductive and Odontostomatological Sciences Federico II University Naples Italy; ^11^ IRCCS Ca' Granda Ospedale Maggiore Policlinico Neurology Unit Milan Italy; ^12^ UOS Neurologia P.O. Fatebenefratelli‐ASST Fatebenefratelli‐Sacco Milan Italy; ^13^ Centro Parkinson e Parkinsonismi Milan Italy; ^14^ IRCCS Ospedale Policlinico San Martino Genoa Italy; ^15^ Parkinson's Disease and Movement Disorders Unit, IRCCS Mondino Foundation Pavia Italy; ^16^ IRCCS San Raffaele Rome Italy; ^17^ IRCCS Istituto Delle Scienze Neurologiche di Bologna Bologna Italy; ^18^ IRCCS SYNLAB SDN Naples Italy; ^19^ University San Raffaele Rome Italy; ^20^ IRCCS San Raffaele Scientific Institute, Neurology Unit Milan Italy; ^21^ SSD Malattie endocrine Diabetologia, ASST Fatebenefratelli‐Sacco Milan Italy; ^22^ Department of Electrical Engineering and Information Technology University Federico II Naples Italy

**Keywords:** gender, levodopa‐naïve, wearing‐off, dyskinesia

## Abstract

**Background:**

Evidence suggests that female gender represents a risk factor for the development of motor/nonmotor fluctuations and dyskinesia in Parkinson's disease (PD). So far, no prospective study has analyzed this aspect in relation to the introduction of levodopa treatment.

**Objective:**

This prospective multicenter study aims to assess the development of motor/nonmotor fluctuations and dyskinesia based on gender over a 2‐year observation period in PD patients starting levodopa.

**Methods:**

Two hundred and eighty‐nine PD patients requiring levodopa at baseline were enrolled at 17 Movement Disorders Centers and followed for 2 years. Gender differences in the development of fluctuations, defined as a score ≥2 in the 19‐item Wearing‐Off Questionnaire, and dyskinesia, defined by Movement Disorders Society Unified Parkinson's Disease Rating Scale Part IV (MDS‐UPDRS‐IV) score >0 on item 4.1 were assessed. Baseline predictors of such complications were evaluated by stepwise multivariate logistic regression analysis.

**Results:**

Two hundred and sixteen patients (139 men, 77 women) completed the follow‐up (M24). By M24, 53,2% of men and 64.9% of women had fluctuations (*P* = 0.048), whereas 5% of men and 14.3% of women developed dyskinesia (*P* = 0.0185). Multivariate analysis showed that female gender significantly predicted wearing‐off (Odds ratio [OR] = 1.930; *P* = 0.0333), whereas older age was a significant protective factor (for 5‐year increase: OR = 0.712; *P* < 0.0001). Multivariate analysis showed that gender (OR = 3.405; *P* = 0.0228) and MDS‐UPDRS Part III score (for a 5‐unit increase: OR = 1.281; *P* = 0.0239) were significant predictors of dyskinesia at M24.

**Conclusions:**

Female gender was the strongest predictor of fluctuations and dyskinesia after 2‐year intake of levodopa. This finding could have important implications for the development of gender‐oriented therapeutic recommendations in early PD.

Prolonged use of levodopa (l‐dopa), the most effective drug in the treatment of Parkinson's disease (PD), can lead to complications, mainly motor/nonmotor fluctuations and dyskinesia, which are one of the main causes of disability among patients.^1^ Predictors of motor/nonmotor fluctuations and dyskinesia are only partially known. Disease duration and l‐dopa treatment (duration and dosage) are among the main determinants.^2^, ^3^

Evidence suggests that female gender may represent a risk factor in the development of motor/nonmotor fluctuations and dyskinesia in PD.^4^, ^5^ So far, we are not aware of any longitudinal studies that have explored this issue in relation to the initiation of l‐dopa treatment.

Our study aimed to collect clinical data on the development of motor/nonmotor fluctuations and dyskinesia in relation to gender over a 2‐year observation period in PD patients starting l‐dopa treatment.

## Patients and Methods

This was a longitudinal national multicenter study, conducted at 17 Italian centers for PD and movement disorders. Patients affected by idiopathic PD were consecutively enrolled in the study if l‐dopa‐naïve and deemed by clinical judgment to require l‐dopa treatment. The protocol was approved by Ethics Committees of all participating centers, and all patients signed informed consent.

Sample size calculation was based on preliminary data obtained from previous trials.^4^, ^5^ Particularly, it has been hypothesized that motor fluctuations (defined as a 19‐item Wearing‐Off Questionnaire [WOQ‐19] score ≥ 2) have a prevalence of 45% in nonexposed men and an OR of 2 for women. Aiming at a 0.05 significance level and a power of 0.80, the estimated sample size based on a *z*‐test was 133 patients per gender, with a men/women ratio of 1. However, according to the epidemiological data reporting that an incidence rate of PD is 1.5–2 times higher in men than in women,^6^ and the design of the study involving consecutive enrollment, we expected a greater number of men to be recruited in the study.

### Objectives and Assessments

The primary objectives of the study were as follows: (1) to describe gender‐related differences in the development of motor/nonmotor fluctuations by analyzing the percentages of women and men who developed such fluctuations, defined as a WOQ‐19 score ≥ 2^7^ assessed at a 2‐year follow‐up after starting l‐dopa treatment; (2) to describe gender‐related differences in the development of dyskinesia, defined as a score > 0 on item 4.1 of the Movement Disorders Society Unified Parkinson's disease rating scale Part IV (MDS‐UPDRS‐IV),^8^ also assessed at a 2‐year follow‐up after starting l‐dopa treatment.

Secondary objectives were to describe gender‐related differences in the development of (1) behavioral complications, assessed by Questionnaire for Impulsive‐Compulsive Disorders in Parkinson's Disease (QUIP),^9^ the Hamilton Depression and the Hamilton Anxiety rating scales (HAM‐D, HAM‐A),^10^, ^11^ the Apathy Evaluation Scale (AES)^12^; (2) nonmotor symptoms assessed by MDS‐UPDRS nonmotor part (Part I) and the nonmotor symptoms severity scale (NMSS)^13^; (3) motor symptoms, assessed by MDS‐UPDRS motor section (Part III); (4) autonomic symptoms assessed by the Scale for Outcomes in Parkinson's disease—Autonomic (SCOPA‐AUT)^14^; (5) global cognitive status assessed by the Montreal cognitive assessment (MoCA)^15^; (6) daily life activities, assessed by MDS‐UPDRS activities of daily living (Part II), the Schwab and England activities of daily living (SEADL)^16^; (7) quality of life, assessed by the 39‐item Parkinson's disease quality of life (PDQ‐39)^17^; and (8) patient's adherence to the therapy, assessed by the Morisky Medication Adherence Scale (MMAS).^18^

### Genetic Substudy

The role of genetic variants in the development of fluctuations and dyskinesia was assessed in a subgroup of patients who accepted to be enrolled in this substudy using an NGS panel of 54 genes, of which 16 are associated with monogenic PD and 38 are risk loci or genes involved in l‐dopa‐induced dyskinesia. The complete list of genes examined is presented in Table S1.

## Statistical Methods

The primary and secondary endpoints were analyzed in the completer population, consisting of all enrolled patients who completed the 24‐month follow‐up.

The co‐primary endpoint, represented by the development of wearing‐off, defined by a WOQ‐19 score ≥2, was analyzed by comparing the two genders on its absolute cumulative frequency during 24 months from the introduction of l‐dopa treatment by means of the χ^2^ test at a nominal statistical significance of 0.05 (one sided). In addition, in the case of a statistically significant result of the above primary analysis, a separate analysis was conducted for the WOQ‐19 motor score ≥1 and WOQ‐19 nonmotor score ≥1 by means of the χ^2^ test at a nominal statistical significance of 0.05 (one sided).

To analyze the co‐primary endpoint, represented by the development of dyskinesia during 24 months from the introduction of l‐dopa as assessed by the MDS‐UPDRS‐IV item 4.1 score >0, we compared the two genders by means of the χ^2^ test at a nominal statistical significance of 0.05 (one sided).

The analyses of the secondary endpoints were performed by means of analysis of covariance (ANCOVA) (in case of a statistically significant parallelism test) models for repeated measures.

In case of statistically significant differences between genders at the univariate analysis, the association between gender and the development of motor/nonmotor fluctuations (WOQ‐19 total score >2, WOQ‐19 motor score ≥1, and WOQ‐19 nonmotor score ≥1) and dyskinesia was evaluated using stepwise multivariate logistic regression models with backward elimination. The “goodness‐of‐fit” was evaluated using Hosmer and Lemeshow tests.^19^ The models were adjusted for baseline characteristics such as age at onset, l‐dopa dosage, concomitant use of monoamine oxidase type B (MAO‐B) inhibitors or dopamine‐agonists, and other clinical features that were significantly associated with the event in the univariate analysis.

## Results

**Fig. 1:**
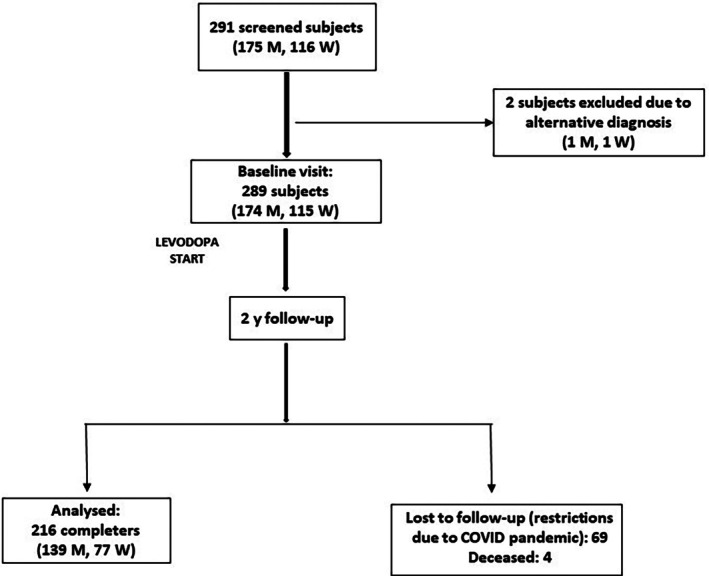
Study flowchart.

A total of 291 patients were asked to take part in the study between February 2019 and November 2022. Figure 1 shows the study flowchart. Two patients ended their clinical workup with a diagnosis of multiple system atrophy (MSA) and iatrogenic parkinsonism (one woman and one man, respectively) and thus were excluded from the study. Thus, 289 patients (174 men and 115 women) underwent baseline examination (see Table S2). A total of 216 patients, 139 (79.9%) men and 77 (67%) women, completed the 24‐month follow‐up and, thus, represent the study cohort. Four patients died during the study. Most dropouts were ascribed to restrictions during the COVID‐19 pandemic, preventing many patients from returning for study assessments.

Baseline characteristics for the completers population are described in Table 1. Men and women were homogeneous for age, age at diagnosis, disease duration, and body mass index (BMI) (Table 1).

**TABLE 1:** Demographic and clinical features based on gender at baseline—completers population

Statistics	Male (n = 139) mean (SD) median (min‐max)	Female (n = 77) mean (SD) median (min‐max)	*P*‐value
Age (y)	63.81 (9.14) 65.00 (39.00; 81.00)	65.34 (9.28) 67.00 (39.00; 86.00)	0.242
Age at diagnosis	63.81 (9.14) 65.00 (39.00; 81.00)	65.34 (9.28) 67.00 (39.00; 86.00)	0.143
Disease duration (mo)	25.75 (24.48) 18.00 (0.00; 168.00)	23.48 (24.00) 13.00 (0.00; 120.00)	0.444
BMI	26.26 (3.58) 26.30 (19.04; 38.31)	25.85 (4.46) 25.39 (18.34; 37.88)	0.497
MDS‐UPDRS I	7.49 (4.68) 7.00 (0.00; 21.00)	8.60 (5.65) 8.00 (1.00; 24.00)	0.126
MDS‐UPDRS II	7.63 (4.56) 7.00 (0.00; 20.00)	8.03 (5.05) 7.00 (1.00; 19.00)	0.559
MDS‐UPDRS III	26.80 (9.35) 26.00 (4.00; 55.00)	27.79 (11.70) 26.00 (6.00; 68.00)	0.526
QUIP	7.18 (9.99) 2.00 (0.00; 46.00)	5.08 (10.13) 1.00 (0.00; 56.00)	0.143
HAM‐A	6.13 (5.25) 5.00 (0.00; 25.00)	8.99 (6.08) 8.00 (0.00; 25.00)	**0.0004**
HAM‐D	5.50 (4.85) 4.00 (0.00; 23.00)	7.54 (4.63) 7.00 (0.00; 24.00)	**0.0032**
AES	32.22 (7.68) 32.00 (18.00; 55.00)	32.31 (8.26) 30.00 (20.00; 55.00)	0.939
SCOPA‐AUT	33.75 (6.58) 34.00 (23.00; 55.00)	37.06 (8.49) 37.00 (23.00; 62.00)	**0.0037**
NMSS	21.45 (20.28) 14.00 (0.00; 84.00)	27.70 (31.07) 15.50 (0.00; 109.00)	0.228
MoCA	24.48 (3.52) 25.00 (15.00; 30.00)	23.51 (4.66) 25.00 (9.00; 29.00)	0.121
SEADL	91.65 (7.95) 90.00 (70.00; 100.00)	89.47 (11.53) 90.00 (40.00; 100.00)	0.145
PDQ‐39 summary index	12.63 (9.29) 10.36 (0.00; 50.16)	17.65 (12.47) 13.23 (1.30; 55.90)	**0.0025**
Morisky scale	7.49 (1.00) 8.00 (3.00; 8.00)	7.37 (1.29) 8.00 (2.50; 8.00)	0.560

*Note*: Significant differences are presented in bold.

Abbreviations: BMI, body mass index; MDS‐UPDRS, Movement Disorders Society Unified Parkinson's Disease Rating Scale; QUIP, Questionnaire for Impulsive‐Compulsive Disorders in Parkinson's Disease; HAM‐A, Hamilton Anxiety rating scale; HAM‐D, Hamilton Depression rating scale; AES, Apathy Evaluation Scale; SCOPA‐AUT, Scale for Outcomes in Parkinson's disease—Autonomic; NMSS, nonmotor symptoms severity scale; MoCA, Montreal cognitive assessment; SEADL, Schwab and England activities of daily living; PDQ‐39, 39‐item Parkinson's disease quality of life.

Mean baseline scores of the HAM‐A and HAM‐D were significantly higher in women (*P* = 0.0004 and *P* = 0.0032, respectively), although scores were within the range of mild severity. Significantly higher scores were also found in women compared to men in the SCOPA‐AUT (*P* = 0.0037), mainly driven by thermoregulatory, pupillomotor and sexual domain, and the summary index of the PDQ‐39 (*P* = 0.0025), mainly driven by mobility, emotional wellness, and bodily discomfort domains. No significant differences were found in other administered scales.

At baseline, 54,6% of men and 46.1% of women were treated with at least one antiparkinsonian drug. Rasagiline was the most prescribed medication, taken in 28.7% of men and 22.6% of women; 36% of men and 33% of women were treated with dopamine agonists. l‐dopa‐equivalent daily dose (LEDD) at baseline was similar in men (122.9 + 127.1 mg/day) and women (109.32 + 129 mg/day; *P* = 0.45). l‐dopa was started after baseline examination in each patient, and the prescribed dose was similar in men (216.7 + 110.1 mg/day) and women (217.1 + 90.8 mg/day, *P* = 0.97).

**Fig. 2:**
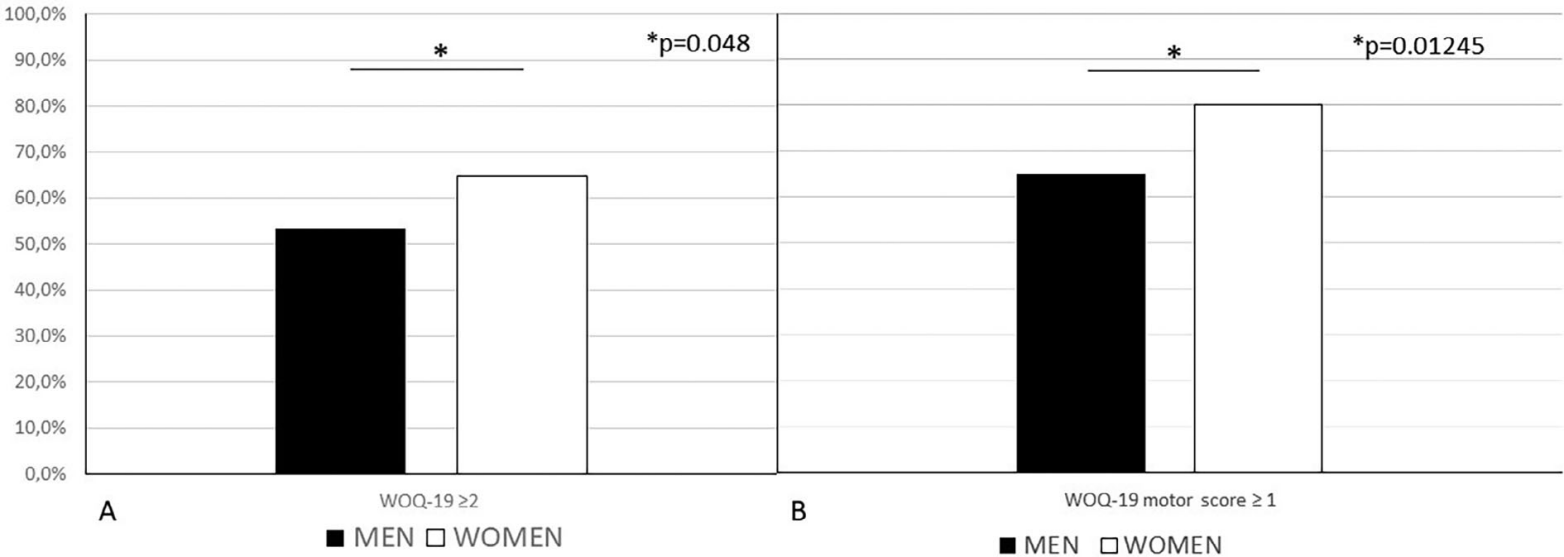
Percentage of patients developing wearing‐off (**A**) and motor fluctuations (**B**) based on gender assessed over a 2‐year observational period since the start of levodopa treatment.

The analysis of the first co‐primary endpoint, represented by a WOQ‐19 score ≥2, showed that 74 (53.2%) men and 50 (64.9%) women had wearing‐off by M24 (Fig. 2A). The difference turned out to be statistically significant (*P* = 0.048).

At the separate analysis of the WOQ‐19 motor score ≥1 and WOQ‐19 nonmotor score ≥1, the results of the χ^2^ test turned out to be statistically significant for the motor score only (*P* = 0.01245). By M24, 80 (65%) men and 60 (80%) women had a motor score ≥1 (Fig. 2B).

No significant association was found between dyskinesia and anxiety in both women and men (*P* = 0.762 and *P* = 0.274, respectively), and between dyskinesia and depression in both women and men (*P* = 0.770 and *P* = 0.274, respectively) by means of χ^2^ test. A trend toward significance was found between motor fluctuations and anxiety in men (*P* = 0.059), whereas no significant association was observed in women (*P* = 0.541). No significant association was found between motor fluctuations and depression in either men (*P* = 0.532) or women (*P* = 0.317) using χ^2^ test.

**Fig. 3:**
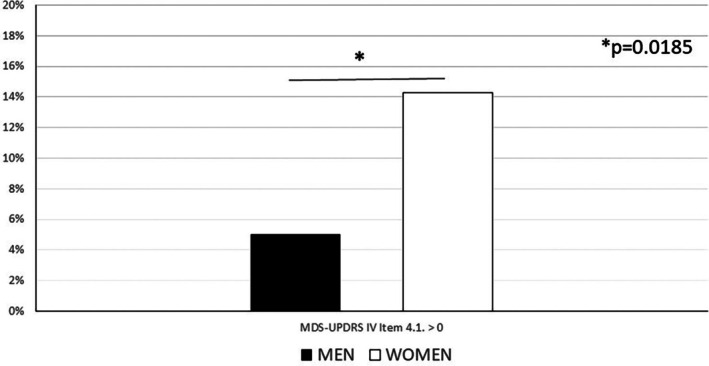
Percentage of patients developing dyskinesia based on gender assessed over a 2‐year observational period since the start of levodopa treatment.

The analysis of the second co‐primary endpoint showed that 7 (5%) men and 11 (14.3%) women developed dyskinesia by M24. The analysis of the MDS‐UPDRS‐IV item 4.1 at M24 showed a statistically significant difference between the two genders (*P* = 0.0185) (Fig. 3).

To assess the interaction between age and gender and l‐dopa dosage and gender, one‐way analysis of variance (ANOVA) was conducted. No significant interaction with gender was found for age (*P* = 0.127) or l‐dopa dosage (*P* = 0.342).

A multivariate logistic regression model was conducted with wearing‐off (yes/no) as the dependent variable. Independent variables included gender (*P* = 0.04795 at the univariate analysis), age (*P* < 0.0001 at the univariate analysis), BMI (*P* = 0.5726 at the univariate analysis, but included in the model as a possible confounding factor in the comparison between the two genders), age at diagnosis (*P* = 0.0001 at the univariate analysis), duration of the disease (in months, *P* = 0.0251 at the univariate analysis), treatment with MAO‐B inhibitors or dopamine agonists (*P* = 0.0105, at the univariate analysis), MDS‐UPDRS Part I score, (*P* = 0.0254 at the univariate analysis), MDS‐UPDRS Part II score (*P* = 0.0060 at the univariate analysis), and MDS‐UPDRS Part III score (*P* = 0.0396 at the univariate analysis).

In the final model the variables significantly associated with the event were gender (OR and 95% Wald confidence interval [CI]: women vs. men: OR = 1.930; 95% CI: 1.062–3.577, *P* = 0.0333), with female gender being a risk factor for wearing‐off, and age (*P* < 0.0001; OR = 0.934, 95% CI: 0.903–0.967 for each year increase; for 5‐year increase: OR = 0.712, 95% CI: 0.597–0.840), with older age being a protective factor (see Table 2). The Hosmer and Lemeshow test turned out to be not statistically significant (*P* = 0.7783).

**TABLE 2:** Significant predictors of wearing‐off and dyskinesia at multivariate analyses

	OR	95% CI	*P*‐value
Wearing‐off as the dependent variable			
Gender	1.930	1.062–3.577	0.0333
Age, each year increase	0.934	0.903–0.967	<0.0001
Age, each 5‐year increase	0.712	0.597–0.840	
Motor wearing‐off as the dependent variable			
Gender	2.52	1.262–5.262	0.0109
Age, each year increase	0.946	0.911–0.983	0.0046
Age, each 5‐year increase	0.759	0.622–0.913	
Dyskinesia as the dependent variable			
Gender	3.405	1.215–10.400	0.0228
MDS‐UPDRS Part III, each unity increase	1.051	1.006–1.098	0.0239
MDS‐UPDRS Part III, each 5‐unit increase	1.281	1.033–1.599	

Abbreviations: OR, Odds ratio; CI, confidence interval; MDS‐UPDRS, Movement Disorders Society Unified Parkinson's Disease Rating Scale.

Furthermore, when the prescribed dose of l‐dopa at baseline was added as a variable to the final model, it turned out not to be significantly associated with the dependent variable (*P* = 0.2159).

A multivariate logistic regression model has been carried out with WOQ‐19 motor score (0/≥1) as the dependent variable, and independent variables included the following: gender (*P* = 0.0249 at the univariate analysis), age (*P* = 0.0061 at the univariate analysis), age at diagnosis (*P* = 0.0114 at the univariate analysis), and treatment with MAO‐B inhibitors or dopamine agonists (*P* = 0.012 at the univariate analysis).

In the final multivariate model, the variables significantly associated with the event were gender (women vs. men: OR 2.52; 95% CI: 1.262–5.262, *P* = 0.0109), with female gender being a risk factor for motor wearing‐off, and age (*P* = 0.0046), with a protective effect (OR = 0.946; 95% CI: 0.911–0.983 for each year increase and OR = 0.759; 95% CI: 0.622–0.913 for each 5‐year increase) (see Table 2).

Furthermore, when the prescribed dose of l‐dopa at baseline was added to the final model, it turned out not to be associated with the dependent variable (*P* = 0.7190).

A multivariate logistic regression model has been carried out with the MDS‐UPDRS IV item 4.1 (0/>0) as the dependent variable and gender (*P* = 0.0185 at the univariate analysis), age (*P* = 0.1066, included for having the estimates adjusted for), BMI (*P* = 0.3544 at the univariate analysis, included as a potential confounder factor in the comparison between the two genders), age at diagnosis (*P* = 0.2267 at the univariate analysis, included for consistency with the previous analysis using the WOQ‐19 ≥ 2 as dependent variable), and MDS‐UPDRS Part III score (*P* = 0.0191 at the univariate analysis) as independent variables.

In the final model, the variables statistically associated with the event were gender (women vs. men: OR = 3.405, 95% CI: 1.215–10.400, *P* = 0.0228) and MDS‐UPDRS Part III score (OR = 1.051, 95% CI: 1.006–1.098 for each unity increase, *P* = 0.0239; OR = 1.281, 95% CI: 1.033–1.599 for each 5‐unit increase), both being risk factors for dyskinesia (see Table 2).

Furthermore, when the prescribed l‐dopa dose at baseline was added to the final model, it turned out not to be associated with the dependent variable (*P* = 0.4634).

### Secondary Endpoints

For QUIP‐RS, the ANCOVA model showed a pattern significantly different between genders overtime (interaction “gender by time” *P* = 0.0272). Indeed, there was an increase from 12 to 24 months in women (least squares means + standard error: 4.5398 + 0.8875 and 5.4629 + 0.8551, respectively) and a decrease in men (least squares means + standard error: 6.251 + 0.66 and 4.0568 + 0.6349, respectively). However, the change between 12 and 24 months in women and in men was not significant, even if we detected a borderline significance level in men (*P* = 0.0545). This borderline significant improvement in men between 12 and 24 months was mainly driven by sex domain and hobbyism‐punding domain. No significant differences between genders overtime were found for other secondary outcomes (see Supporting Information).

### Genetic Substudy

A total of 142 patients (89 men, 53 women) enrolled in the genetic substudy completed the 2‐year follow‐up. Detailed genetic findings in patients participating in the substudy are presented in Supplementary Tables S1 and S3. A total of 310 gene variants, 187 in men and 123 in women, were found. The prevalence of genetic variants did not differ between genders (*P* = 0.543) and was not associated with wearing‐off or dyskinesia at the 2‐year follow‐up in the univariate analysis (*P* = 0.482 and *P* = 0.294, respectively).

### Antiparkinsonian Medications at Follow‐Up and Safety

l‐dopa daily dose at the 1‐year follow‐up was similar between men and women (263 + 111.5 vs. 243.1 + 84.3 mg/day; *P* = 0.186). LEDD, excluding l‐dopa, at the 1‐year follow‐up was similar between men and women (133.8 + 120.3 vs. 106.8 + 119.7; *P* = 0.122). l‐dopa daily dose at the 2‐year follow‐up was slightly higher in men compared to women (327.6 + 148 vs. 286.7 + 125.6 mg/day; *P* = 0.041), whereas LEDD, excluding levodopa, at the 2‐year follow‐up was similar between men and women (139.66 + 138.2 vs. 114.9 + 121; *P* = 0.191).

Adverse events and serious adverse events are provided in Tables S4 and S5.

In particular, 14.4% of men and 21.7% of women reported experiencing at least one adverse event during the study.

## Discussion

This is the first study designed to longitudinally evaluate the development of fluctuations and dyskinesia and their predictors in men and women with PD enrolled at their first ever intake of l‐dopa.

At the 2‐year follow‐up, we found that wearing‐off was significantly more common in women than men. Moreover, in line with previous cross‐sectional studies assessing fluctuations using WOQ‐19,^20^, ^21^ we found that WOQ‐19 was very sensitive in detecting wearing‐off.

The early recognition of wearing‐off is critical to optimize disease treatment and improve long‐term clinical outcomes. Consistent with our results, a previous cross‐sectional multicenter study using WOQ‐19 in a larger PD population, who had been treated with l‐dopa and/or dopamine agonist for >1 year before the study screening, found a significantly higher prevalence of wearing‐off in women compared to men.^5^, ^21^ A higher prevalence of wearing‐off in women has also been demonstrated using WOQ‐9, and a shorter time latency to develop motor fluctuations has been shown in women compared to men.^22^, ^23^

We found that female gender was the main predictor of fluctuations with an OR of 1.930, whereas older age was a protective factor with an OR of 0.712 for each 5‐years increase. Female gender was the main predictor of motor fluctuations with an OR of 2.52, whereas older age confirmed to be a protective factor with an OR of 0.759 for each 5‐years increase. Both findings are consistent with data from a large cross‐sectional study using WOQ‐19 score, showing that women had an 80.1% higher risk of experiencing wearing‐off than men, and every increase of 1 year of age was associated with a 5% risk reduction of wearing‐off.^21^ Young age and female gender were significant predictors of wearing‐off also in the STRIDE‐PD study, a prospective, multicenter double‐blind study comparing l‐dopa/carbidopa versus l‐dopa/carbidopa/entacapone in early PD.^24^ Conversely, some previous studies assessing longitudinal PD cohorts did not find differences in the risk of wearing‐off between genders,^25^, ^26^, ^27^ likely due to different designs, inclusion criteria, and measures for wearing‐off compared to our study. This study advances our knowledge on the topic and suggests that early detection of wearing‐off is advisable, particularly in women, to manage promptly this debilitating phenomenon.

At the 2‐year follow‐up, we also found that the prevalence of dyskinesia was significantly higher in women than men. This finding is consistent with previous retrospective and prospective studies.^25^, ^26^, ^28^ Indeed, female gender has been associated with a shorter time to occurrence of l‐dopa‐induced dyskinesia with a median time to dyskinesia of 4 years in women and 6 years in men.^29^

Moreover, female gender was a very strong predictor of dyskinesia (OR: 3.405), whereas motor severity at baseline, assessed by MDS‐UPDRS Part III, was a less strong predictor of dyskinesia with an OR of 1.281 for each 5‐unit increase. Again, the l‐dopa dose at baseline did not predict dyskinesia in our cohort. This finding is consistent with results from a large Italian PD cohort showing that l‐dopa daily intake was not associated with the risk of dyskinesia, whereas female gender showed a 39% higher risk of dyskinesia.^30^ A multivariate analysis from the PPMI cohort assessing 423 PD patients with a median follow‐up duration of 4.6 years also reported female gender among the most important predictors of dyskinesia.^31^ This is the first study showing such a significant gender effect on dyskinesia at a very early stage after the introduction of l‐dopa and could push toward a change in clinical practice, considering gender among the main determinants of therapeutic choices rather than using a one‐size‐fits‐all approach.

BMI did not predict dyskinesia nor fluctuations, strongly supporting the idea that female gender per se is an independent predictor of motor complications. This finding is consistent with a previous population‐based 5‐year longitudinal study recruiting drug‐naïve PD patients, where female gender resulted to be a risk factor for dyskinesia regardless of body weight.^25^

We have recently reported a greater bioavailability of l‐dopa in l‐dopa‐naïve women with PD compared to men. In this study, l‐dopa pharmacokinetics was assessed at the first ever l‐dopa intake of l‐dopa/benserazide (100/25 mg), and women showed significantly higher area under curve and maximum plasma concentration than men irrespective of body weight.^32^ The same findings were also reported in pharmacokinetics studies assessing patients on chronic l‐dopa treatment.^33^, ^34^ Possible differences in metabolic l‐dopa pathways have been suggested, as women have about 25% lower catechol‐*O*‐methyltransferase enzyme activity than men,^35^ but differences in gastrointestinal transit times could also be involved.

We suggest that in our cohort l‐dopa dosage did not emerge as a significant predictor of fluctuations and dyskinesia due to the similar dosages prescribed in both genders and the short duration of administration. The observation that only at the end of the observation period women received a l‐dopa dose slightly lower than men may reflect the attention paid by neurologists toward the development of dyskinesia in women, as we did not find significant differences between genders overtime on MDS‐UPDRS Part II and Part III scores.

We had no evidence of a genetic contribution to the gender‐related differences in the development of fluctuations and dyskinesia. Consistent with these findings, previous studies excluded that SNCA variants increasing the expression of SNCA gene predicted dyskinesia or motor fluctuations in PD.^30^, ^36^ Similarly, GBA mutations were not found to be associated with a higher risk of developing motor complications in different PD populations.^37^, ^38^ Finally, dopamine receptor D2 gene polymorphisms were shown to provide a strong protection toward dyskinesia only in men and not in women.^39^

Hormonal factors may contribute to biological differences in the expression of PD, with a lower incidence and a more benign phenotype at onset in women, mainly related to the estrogenic status.^40^ However, once the disease has started, shorter time to develop wearing‐off and dyskinesia in women argues against a persistent protective effect of estrogens. Sex‐chromosome genes and sexual dimorphism in brain structure and function are also supposed to have a role in determining gender differences in PD.^41^, ^42^

One limitation of our study is that only Italian patients were enrolled; therefore, our findings are not widely generalizable and should be confirmed particularly in non‐Caucasian populations. Moreover, the high number of dropouts poses a risk of bias in the study results, but it was not preventable due to the COVID pandemic.

We demonstrated that female gender was the most important predictor of fluctuations and dyskinesia in early PD patients after a 2‐year intake of l‐dopa. Overall, our findings could have important implications in early disease stages and foster the need for the development of gender‐oriented therapeutic recommendations.

## Author Roles

(1) Research Project: A. Conception, B. Organization, C. Execution; (2) Statistical Analysis: A. Design, B. Execution, C. Review and Critique; (3) Manuscript Preparation: A. Writing of the First Draft, B. Review and Critique.

M.T.P.:1A, 1B, 1C, 2A, 2B, 3A

M.P.: 1A, 1B, 1C, 2A, 3B

M.C.R.:1C, 3B

M.A.:1C, 3B

G.A.:1C, 3B

L.A.:1C, 3B

P.B.:1C, 3B

M.C.:1C, 3B

R.C.:1C, 3B

E.C.:1C, 3B

R.C.:1C, 3B

I.C.:1C, 3B

G.D.:1C, 3B

R.D.M.:1C, 3B

A.D.R.:1C, 3B

A.D.F.:1C, 3B

R.E.:1C, 3B

V.F.:1C, 3B

A.G.:1C, 3B

M.G.:1C, 3B

F.I.:1C, 3B

G.L.:1C, 3B

R.M.:1C, 3B

C.P.:1C, 3B

M.P.:1C, 3B

F.R.:1C, 3B

C.S.:1C, 3B

F.S.:1C, 3B

A.T.:1C, 3B

L.V.:1C, 3B

M.A.V.:1C, 3B

G.V.:1C, 3B

R.Z.:1C, 3B

A.S.:1C, 3B

F.A.:1C, 3B

C.R.:1C, 3B

M.R.:1C, 3B

B.G.:1C, 2C, 3A

A.N.:1C, 2C, 3A

A.C.:1C, 2C, 3A

C.R.:1C, 2C, 3A

C.P.:1C, 2C, 3A

## Disclosures

**Ethical Compliance Statement:** The study was approved by Comitato Etico Campania Sud (*P*.U. n. 4 obtained on February 4, 2019). Written informed consent was obtained for each enrolled patient. We confirm that we have read the journal's position on issues involved in ethical publication and affirm that this work is consistent with those guidelines.

**Funding Sources and Conflict of Interest:** This study was funded by AIFA, Italian Medicines Agency (grant: AIFA‐2016‐02364714).

The authors declare that they have no conflict of interest relevant to this work.

**Financial Disclosures for the Previous 12 Months:** The authors declare that there are no additional disclosures to report.

## Supporting information


**Data S1.** Additional results from statistical analyses of secondary outcomes.

**Table S1.** Panel of genes examined with number of variants found based on gender.

**Table S2.** Baseline characteristics by gender and overall—all included patients.

**Table S3.** Detailed genetic findings in patients participating in the substudy (89 men, 53 women) based on the genes examined.

**Table S4.** Absolute and percentage frequencies of patients with at least one adverse event throughout the study by gender and overall—all included patients.

**Table S5.** Absolute and percentage frequencies of patients with at least one serious adverse event throughout the study by gender and overall—all included patients.

## Data Availability

The data that support the findings of this study are available on request from the corresponding author. The data are not publicly available due to privacy or ethical restrictions.

## References

[1] Lang AE , Lees A . Management of Parkinson's disease: an evidence‐based review. Mov Disord 2002;17:S1–S166. 10.1002/mds.5555. 12211134

[2] Sharma JC , Bachmann CG , Linazasoro G . Classifying risk factors for dyskinesia in Parkinson's disease. Parkinsonism Relat Disord 2010;16:490–497. 10.1016/j.parkreldis.2010.06.003. 20598622

[3] Cilia R , Akpalu A , Sarfo FS , et al. The modern pre‐levodopa era of Parkinson's disease: insights into motor complications from sub‐Saharan Africa. Brain 2014;137:2731–2742. 10.1093/brain/awu195. 25034897 PMC4163032

[4] Picillo M , Palladino R , Moccia M , et al. Gender and non‐motor fluctuations in Parkinson's disease: a prospective study. Parkinsonism Relat Disord 2016;27:89–92. 10.1016/j.parkreldis.2016.04.001. 27066847

[5] Colombo D , Abbruzzese G , Antonini A , et al. The “gender factor” in wearing‐off among patients with Parkinson's disease: a post hoc analysis of DEEP study. Scientific World Journal 2015;2015:787451. 10.1155/2015/787451. 25685848 PMC4320843

[6] Hirsch L , Jette N , Frolkis A , Steeves T , Pringsheim T . The incidence of Parkinson's disease: a systematic review and meta‐analysis. Neuroepidemiology 2016;46(4):292–300. 10.1159/000445751. 27105081

[7] Stacy M , Hauser R . Development of a patient questionnaire to facilitate recognition of motor and non‐motor wearing‐off in Parkinson's disease. J Neural Transm 2007;114:211–217. 10.1007/s00702-006-0554-y. 16897594

[8] Goetz CG , Tilley BC , Shaftman SR , et al. Movement Disorder Society‐sponsored revision of the unified Parkinson's disease rating scale (MDS‐UPDRS): scale presentation and clinimetric testing results. Mov Disord 2008;23:2129–2170. 10.1002/mds.22340. 19025984

[9] Weintraub D , Mamikonyan E , Papay K , Shea JA , Xie SX , Siderowf A . Questionnaire for impulsive‐compulsive disorders in Parkinson's disease‐rating scale. Mov Disord 2012;27:242–247. 10.1002/mds.24023. 22134954 PMC3537263

[10] Hamilton M . Development of a rating scale for primary depressive illness. Br J Soc Clin Psychol 1967;6:278–296. 10.1111/j.2044-8260.1967.tb00530.x. 6080235

[11] Hamilton M . The assessment of anxiety states by rating. Br J Med Psychol 1959;32:50–55. 10.1111/j.2044-8341.1959.tb00467.x. 13638508

[12] Marin RS , Biedrzycki RC , Firinciogullari S . Reliability and validity of the apathy evaluation scale. Psychiatry Res 1991;38:143–162. 10.1016/0165-1781(91)90040-v. 1754629

[13] Martinez‐Martin P , Rodriguez‐Blazquez C , Abe K , et al. International study on the psychometric attributes of the non‐motor symptoms scale in Parkinson disease. Neurology 2009;73:1584–1591. 10.1212/WNL.0b013e3181c0d416. 19901251

[14] Visser M , Marinus J , Stiggelbout AM , van Hilten JJ . Assessment of autonomic dysfunction in Parkinson's disease: the SCOPA‐AUT. Mov Disord 2004;19:1306–1312. 10.1002/mds.20153. 15390007

[15] Nasreddine ZS , Phillips NA , Bédirian V , et al. The Montreal cognitive assessment, MoCA: a brief screening tool for mild cognitive impairment. J Am Geriatr Soc 2005;53:695–699. 10.1111/j.1532-5415.2005.53221.x. 15817019

[16] Gilligham FJ . Schwab and England activities of daily living. Third Symp of Parkinson's Disease. Scotland: E. & S. Livingstone Ltd; 1969:152–157.

[17] Peto V , Jenkinson C , Fitzpatrick R , Greenhall R . The development and validation of a short measure of functioning and well being for individuals with Parkinson's disease. Qual Life Res 1995;4:241–248. 10.1007/BF02260863. 7613534

[18] Morisky DE , Ang A , Krousel‐Wood M , Ward HJ . Predictive validity of a medication adherence measure in an outpatient setting. J Clinical Hypertension 2008;10:348–354. 10.1111/j.1751-7176.2008.07572.x. PMC2562622 18453793

[19] Hosmer DW , Lemeshow S . Applied Logistic Regression. 2nd ed. Hoboken, New Jersey, USA: John Wiley & Sons, Inc; 2000.

[20] Silburn PA , Mellick GD , Vieira BI , Danta G , Boyle RS , Herawati L . Utility of a patient survey in identifying fluctuations in early stage Parkinson's disease. J Clin Neurosci 2008;15:1235–1239. 10.1016/j.jocn.2007.09.018. 18824360

[21] Stocchi F , Antonini A , Barone P , et al. Early DEtection of wearing‐off in Parkinson disease: the DEEP study. Parkinsonism Relat Disord 2014;20:204–211. 10.1016/j.parkreldis.2013.10.027. 24275586

[22] Stacy MA , Murck H , Kroenke K . Responsiveness of motor and nonmotor symptoms of Parkinson disease to dopaminergic therapy. Prog Neuropsychopharmacol Biol Psychiatry 2010;34:57–61. 10.1016/j.pnpbp.2009.09.023. 19793544

[23] Sato K , Hatano T , Yamashiro K , et al. Prognosis of Parkinson's disease: time to stage III, IV, V, and to motor fluctuations. Mov Disord 2006;21:1384–1395. 10.1002/mds.20993. 16763980

[24] Olanow WC , Kieburtz K , Rascol O , et al. Factors predictive of the development of levodopa‐induced dyskinesia and wearing‐off in Parkinson's disease. Mov Disord 2013;28:1064–1071. 10.1002/mds.25364. 23630119

[25] Bjornestad A , Forsaa EB , Pedersen KF , Tysnes OB , Larsen JP , Alves G . Risk and course of motor complications in a population‐based incident Parkinson's disease cohort. Parkinsonism Relat Disord 2016;22:48–53. 10.1016/j.parkreldis.2015.11.007. 26585090

[26] Iwaki H , Blauwendraat C , Leonard HL , et al. Differences in the presentation and progression of Parkinson's disease by sex. Mov Disord 2021;36:106–117. 10.1002/mds.28312. 33002231 PMC7883324

[27] Kelly MJ , Lawton MA , Baig F , et al. Predictors of motor complications in early Parkinson's disease: a prospective cohort study. Mov Disord 2019;34:1174–1183. 10.1002/mds.27783. 31283854 PMC6771533

[28] Jeong SH , Lee HS , Lee PH , Sohn YH , Chung SJ . Does dopamine deficiency affect sex‐dependent prognosis in Parkinson's disease? Parkinsonism Relat Disord 2022;102:57–63. 10.1016/j.parkreldis.2022.07.012. 35961198

[29] Hassin‐Baer S , Molchadski I , Cohen OS , et al. Gender effect on time to levodopa‐induced dyskinesias. J Neurol 2011;258:2048–2053. 10.1007/s00415-011-6067-0. 21533825

[30] Tirozzi A , Modugno N , Palomba NP , et al. Analysis of genetic and non‐genetic predictors of levodopa induced dyskinesia in Parkinson's disease. Front Pharmacol 2021;12:640603. 10.3389/fphar.2021.640603. 33995045 PMC8118664

[31] Eusebi P , Romoli M , Paoletti FP , Tambasco N , Calabresi P , Parnetti L . Risk factors of levodopa‐induced dyskinesia in Parkinson's disease: results from the PPMI cohort. NPJ Parkinsons Dis 2018;4:33. 10.1038/s41531-018-0069-x. 30480086 PMC6240081

[32] Conti V , Izzo V , Russillo MC , et al. Gender differences in levodopa pharmacokinetics in levodopa‐naïve patients with Parkinson's disease. Front Med 2022;9:909936. 10.3389/fmed.2022.909936. PMC9193593 35712091

[33] Kumagai T , Nagayama H , Ota T , Nishiyama Y , Mishina M , Ueda M . Sex differences in the pharmacokinetics of levodopa in elderly patients with Parkinson disease. Clin Neuropharmacol 2014;37:173–176. 10.1097/WNF.0000000000000051. 25384078

[34] Contin M , Lopane G , Belotti LMB , Galletti M , Cortelli P , Calandra‐Buonaura G . Sex is the main determinant of levodopa clinical pharmacokinetics: evidence from a large series of levodopa therapeutic monitoring. J Parkinsons Dis 2022;12(8):2519–2530. 10.3233/JPD-223374. 36373294 PMC9837688

[35] Boudíková B , Szumlanski C , Maidak B , Weinshilboum R . Human liver catechol‐O‐methyltransferase pharmacogenetics. Clin Pharmacol Ther 1990;48:381–389. 10.1038/clpt.1990.166. 2225698

[36] Corrado L , de Marchi F , Tunesi S , et al. The length of *SNCA* Rep1 microsatellite may influence cognitive evolution in Parkinson's disease. Front Neurol 2018;9:213. 10.3389/fneur.2018.00213. 29662465 PMC5890103

[37] Maple‐Grødem J , Paul KC , Dalen I , et al. Lack of association between GBA mutations and motor complications in European and American Parkinson's disease cohorts. J Parkinsons Dis 2021;11:1569–1578. 10.3233/JPD-212657. 34275908 PMC8609705

[38] Oeda T , Umemura A , Mori Y , et al. Impact of glucocerebrosidase mutations on motor and nonmotor complications in Parkinson's disease. Neurobiol Aging 2015;36:3306–3313. 10.1016/j.neurobiolaging.2015.08.027. 26422360

[39] Zappia M , Annesi G , Nicoletti G , et al. Sex differences in clinical and genetic determinants of levodopa peak‐dose dyskinesias in Parkinson disease: an exploratory study. Arch Neurol 2005;62:601–605. 10.1001/archneur.62.4.601. 15824260

[40] Reale C , Invernizzi F , Panteghini C , Garavaglia B . Genetics, sex, and gender. J Neurosci Res 2023;101:553–562. 10.1002/jnr.24945. 34498752

[41] Russillo MC , Andreozzi V , Erro R , et al. Sex differences in Parkinson's disease: from bench to bedside. Brain Sci 2022;12:917. 10.3390/brainsci12070917. 35884724 PMC9313069

[42] Picillo M , LaFontant DE , Bressman S , et al. Sex‐related longitudinal change of motor, non‐motor, and biological features in early Parkinson's disease. J Parkinsons Dis 2022;12(1):421–436. 10.3233/JPD-212892. 34744052 PMC8842783

