# Supplementary material for: Gender Is the Main Predictor of Wearing‐Off and Dyskinesia in Levodopa‐Naïve Patients with Parkinson's Disease
Source: Mov Disord Clin Pract. 2025 May 29;12(11):1774–83. doi: 10.1002/mdc3.70143 (PMC12625146; doi:10.1002/mdc3.70143)
Supplement: Supplementary file 1 — Data S1. Additional results from statistical analyses of secondary outcomes. [file MDC3-12-1774-s004.docx]

**Supplementary data**

Secondary outcomes:

As for MDS-UPDRS Part I, the ANCOVA model did not show significantly different pattern of the two genders on the considered times (interaction "gender by time" P = 0.1303).

With regard to MDS-UPDRS Part II, ANOVA model did not show statistically different pattern of the two genders on the considered times (interaction "gender by time" P = 0.2172).

Regarding MDS-UPDRS Part III, the ANCOVA model did not show statistically different of the two genders on the considered times (interaction "gender by time" P = 0.0819).

As regards HAM-A, ANCOVA model did not show statistically different pattern of the two genders on the considered times (interaction "gender by time" P = 0.8600).

As for HAM-D, ANCOVA model did not show significantly different pattern of the two genders on the considered times (interaction "gender by time" P = 0.8194).

Regarding AES, ANCOVA model did not show statistically different pattern of the two genders on the considered times (interaction "gender by time" P = 0.3157).

As for AES caregiver-rated, ANCOVA model did not show significantly different pattern of the two genders on the considered times (interaction "gender by time" P = 0.3514).

As regards SCOPA-AUT, ANCOVA model did not show statistically different pattern of the two genders on the considered times (interaction "gender by time" P = 0.1914).

As for NMSS, ANCOVA model did not show statistically different pattern of the two genders on the considered times (interaction "gender by time" P = 0.2589).

As for MoCA, ANCOVA model did not show statistically different pattern of the two genders on the considered times (interaction "gender by time" P = 0.5465).

As regards SEADL, ANCOVA model did not show statistically different pattern of the two genders on the considered times (interaction "gender by time" P = 0.9366).

As for PDQ-39, ANCOVA model did not show statistically different pattern of the two genders on the considered times (interaction "gender by time" P = 0.1363).

Finally, as regards Moriski scale, ANCOVA model did not show statistically different pattern of the two genders on the considered times (interaction "gender by time" P = 0.2589).
