# Supplementary material for: Gender Is the Main Predictor of Wearing‐Off and Dyskinesia in Levodopa‐Naïve Patients with Parkinson's Disease
Source: Mov Disord Clin Pract. 2025 May 29;12(11):1774–83. doi: 10.1002/mdc3.70143 (PMC12625146; doi:10.1002/mdc3.70143)
Supplement: Supplementary file 2 — Table S1. Panel of genes examined with number of variants found based on gender. [file MDC3-12-1774-s003.docx]

Supplementary Table 1: Panel of genes examined with number of variants found according to gender

| **GENE SYMBOL** | **GENE FULL NAME** | **GENE ALIAS** | **Total number of variants/Gender distribution** |
| --- | --- | --- | --- |
| ADORA2A | adenosine A2a receptor | A2aR; RDC8; ADORA2 | 1M |
| ANK2 | ankyrin 2 | LQT4; ANK-2; FAP87; CFAP87; brank-2 | 25 (17M/8F) |
| ATP13A2 | ATPase cation transporting 13A2 | CLN12; KRPPD; PARK9; SPG78; HSA9947 | 8 (5M/3F) |
| BDNF | brain derived neurotrophic factor | ANON2; BULN2 | none |
| C5orf24 | chromosome 5 open reading frame 24 | - | 1M |
| CHCHD2 | coiled-coil-helix-coiled-coil-helix domain containing 2 | MNRR1; NS2TP; MIX17B; PARK22; C7orf17 | none |
| COMT | catechol-O-methyltransferase | HEL-S-98n | 2 (1M/1F) |
| CTSB | cathepsin B | APPS; CPSB; RECEUP | 3 (2M/1F) |
| DNAH1 | dynein axonemal heavy chain 1 | HL11; HDHC7; HL-11; CILD37; DNAHC1; HSRF-1; SPGF18; XLHSRF-1 | 23 (16M/7F) |
| DNAJC13 | DnaJ heat shock protein family (Hsp40) member C13 | RME8; PARK21 | 12 (6M/6F) |
| DNAJC6 | DnaJ heat shock protein family (Hsp40) member C6 | DJC6; PARK19 | 8 (4M/3F)* |
| DRD2 | dopamine receptor D2 | D2R; D2DR | None |
| DRD3 | dopamine receptor D3 | D3DR; ETM1; FET1 | None |
| DYRK1A | dual specificity tyrosine phosphorylation regulated kinase 1A | MNB; DYRK; HP86; MNBH; MRD7; DYRK1 | 4 (3M/1F) |
| FBRSL1 | fibrosin like 1 | - | 13 (8M/4F)* |
| FBXO7 | F-box protein 7 | FBX; FBX7; PKPS; FBX07; PARK15 | 4 (2M/2F) |
| FGF20 | fibroblast growth factor 20 | RHDA2; FGF-20 | None |
| FYN | FYN proto-oncogene, Src family tyrosine kinase | SLK; SYN; p59-FYN | 2 (1M/1F) |
| GALC | galactosylceramidase | - | 10 (7M/3F) |
| GALNT14 | polypeptide N-acetylgalactosaminyltransferase 14 | GALNT15; GalNac-T10; GalNac-T14 | 2 (1M/1F) |
| GBA | glucosylceramidase beta | GCB; GBA1; GLUC | 16 (9M/ 7F)* |
| GBF1 | golgi brefeldin A resistant guanine nucleotide exchange factor 1 | CMT2GG; ARF1GEF | 12 (8M/ 4F) |
| GCH1 | GTP cyclohydrolase 1 | GCH; DYT5; DYT14; DYT5a; GTPCH1; HPABH4B; GTP-CH-1 | 2 (1M/1F) |
| GRIN2A | glutamate ionotropic receptor NMDA type subunit 2A | LKS; EPND; FESD; NR2A; GluN2A; NMDAR2A | 7 (5M/1F)* |
| ITPKB | inositol-trisphosphate 3-kinase B | IP3K; IP3KB; PIG37; IP3K-B; IP3-3KB | 8 (7M/1F) |
| LAMP2 | lysosomal associated membrane protein 2 | DND; LAMPB; CD107b; LAMP-2; LGP-96; LGP110 | 1F |
| LRRK2 | leucine rich repeat kinase 2 | PARK8; RIPK7; ROCO2; AURA17; DARDARIN | 17 (9M/8F) |
| MAD2L2 | mitotic arrest deficient 2 like 2 | REV7; FANCV; MAD2B; POLZ2 | 1M |
| MAOB | monoamine oxidase B | - | 1F |
| MAP7 | microtubule associated protein 7 | EMAP115; E-MAP-115 | 9 (7M/2F) |
| MAPT | microtubule associated protein tau | TAU; MSTD; PPND; DDPAC; MAPTL; MTBT1; MTBT2; tau-40; FTDP-17; PPP1R103 | 2M |
| MBNL2 | muscleblind like splicing regulator 2 | MBLL; MBLL39; PRO2032 | 2M |
| NIBAN2 (FAM29B) | niban apoptosis regulator 2 | OC58; MEG-3; C9orf88; FAM129B; MINERVA; bA356B19.6 | 6 (2M/3F)* |
| NOD2 | nucleotide binding oligomerization domain containing 2 | CD; ACUG; BLAU; IBD1; YAOS; BLAUS; NLRC2; NOD2B; CARD15; CLR16.3; PSORAS1 | 16 (9M/ 7F) |
| OPRK1 | opioid receptor kappa 1 | KOP; KOR; KOR1; OPRK; KOR-1; K-OR-1 | 3 (2M/1F) |
| OPRM1 | opioid receptor mu 1 | MOP; MOR; LMOR; MOR1; OPRM; M-OR-1 | 7 (3M/4F) |
| PARK7 | Parkinsonism associated deglycase | DJ1; DJ-1; GATD2; HEL-S-67p | 4 (3M/1F) |
| PINK1 | PTEN induced kinase 1 | BRPK; PARK6 | None |
| PRKN | parkin RBR E3 ubiquitin protein ligase | PDJ; AR-JP; LPRS2; PARK2 | 4 (1M/3F) |
| PTRHD1 | peptidyl-tRNA hydrolase domain containing 1 | C2orf79 | None |
| RAB39B | RAB39B, member RAS oncogene family | WSN; BGMR; WSMN; MRX72; XLID72 | None |
| RABGEF1 | RABGEF1 pseudogene 1 | GS1-124K5.11 | None |
| SH3GL2 | SH3 domain containing GRB2 like 2, endophilin A1 | CNSA2; SH3P4; EEN-B1; SH3D2A | 1F |
| SIPA1L2 | signal induced proliferation associated 1 like 2 | SPAL2; SPAR2 | 9 (6M/2F)* |
| SLC6A3 | solute carrier family 6  member 3 | DAT; DAT1; PKDYS; PKDYS1 | 1F |
| SNCA | synuclein alpha | PD1; NACP; PARK1; PARK4 | None |
| STAB1 | stabilin 1 | FEX1; FEEL1; FEEL-1; FELE-1; SCARH2; STAB-1; CLEVER-1 | 24 (10M/14F) |
| SV2C | synaptic vesicle glycoprotein 2C | - | 1M |
| SYNJ1 | synaptojanin 1 | DEE53; EIEE53; INPP5G; PARK20 | 4 (1M/3F) |
| TMEM175 | transmembrane protein 175 | hTMEM175 | 4 (3M/1F) |
| TMEM230 | transmembrane protein 230 | HSPC274; C20orf30; dJ1116H23.2.1 | 3 (2M/1F) |
| VAMP4 | vesicle associated membrane protein 4 | VAMP-4; VAMP24 | none |
| VPS13C | vacuolar protein sorting 13 homolog C | PARK23 | 21 (11M/10F)* |
| VPS35 | VPS35 retromer complex component | MEM3; PARK17 | 2 (1M/1F) |
| * 2 variants in the same patient | | | |

All patients’ DNA was extracted from whole blood’s lymphocytes and enriched with Illumina DNA Prep with Enrichment protocol using xGen IDT probes. The amplified libraries were sequenced on the Illumina Miseq platform. The generated reads were aligned to human genome assembly hg19 (Human Feb. 2009 GRCh37) and the identified variants were annotated (eVai software, enGenome) and filtered, focusing on rare variants (minimum allele frequency <1% in GnomAD browser “https://gnomad.broadinstitute.org/”).
