# Supplementary material for: Gender Is the Main Predictor of Wearing‐Off and Dyskinesia in Levodopa‐Naïve Patients with Parkinson's Disease
Source: Mov Disord Clin Pract. 2025 May 29;12(11):1774–83. doi: 10.1002/mdc3.70143 (PMC12625146; doi:10.1002/mdc3.70143)
Supplement: Supplementary file 3 — Table S2. Baseline characteristics by gender and overall—all included patients. [file MDC3-12-1774-s002.docx]

**Supplementary Table 2: Baseline characteristics by gender and overall – All included patients**

_____________________________________________________________________________________________________________________________________

Male Female ALL

Statistics (N=174) (N=115) (N=289)

_____________________________________________________________________________________________________________________________________

Age

n 174 115 289

Mean (SD) 64.72 (9.49) 66.10 (9.41) 65.27 (9.46)

Median (Min-Max) 66.00 (39.00;82.00) 67.00 (39.00;87.00) 67.00 (39.00;87.00)

BMI

n 146 97 243

Mean (SD) 26.25 (3.68) 26.10 (5.06) 26.19 (4.28)

Median (Min-Max) 26.31 (18.31;38.31) 25.24 (18.34;45.66) 25.88 (18.31;45.66)

Age at diagnosis

n 174 115 289

Mean (SD) 64.72 (9.49) 66.10 (9.41) 65.27 (9.46)

Median (Min-Max) 66.00 (39.00;82.00) 67.00 (39.00;87.00) 67.00 (39.00;87.00)

Disease duration

(months) n 172 115 287

Mean (SD) 25.52 (23.93) 25.84 (31.20) 25.65 (27.02)

Median (Min-Max) 18.50 (0.00;168.00) 16.00 (0.00;252.00) 18.00 (0.00;252.00)
