# Supplementary material for: Gender Is the Main Predictor of Wearing‐Off and Dyskinesia in Levodopa‐Naïve Patients with Parkinson's Disease
Source: Mov Disord Clin Pract. 2025 May 29;12(11):1774–83. doi: 10.1002/mdc3.70143 (PMC12625146; doi:10.1002/mdc3.70143)
Supplement: Supplementary file 4 — Table S3. Detailed genetic findings in patients participating in the substudy (89 men, 53 women) based on the genes examined. [file MDC3-12-1774-s005.docx]

Supplementary Table 3: Detailed genetic findings in patients participating to the substudy ((89 men, 53 women) according to genes examined.

| Gene | Sex | Chromosomic position | HGVSc | HGVSp | ACMG (eVai) |
| --- | --- | --- | --- | --- | --- |
| ADORA2A | M | chr22:24837321G/C | c.1103G>C | p.Gly368Ala | VUS |
| ANK2 | M | chr4:114278820G/A | c.9046G>A | p.Glu3016Lys | Likely Benign |
|  | F | chr4:114276526C/G | c.6752C>G | p.Pro2251Arg | VUS |
|  | M | chr4:114284618T/G | c.10881T>G | p.His3627Gln | Likely Benign |
|  | M | chr4:114276922C/T | c.7148C>T | p.Pro2383Leu | Likely Benign |
|  | M | chr4:114214588C/T | NM_001148.4:c.2377-8C>T |  | Benign |
|  | M | chr4:114275344T/A | c.55070T>A | p.Val1857Glu | VUS |
|  | M | chr4:114278820G/A | c.9046G>A | p.Glu3016Lys | Likely Benign |
|  | M | chr4:114275283 G/A | c.5509G>A | p.Ala1837Thr | Likely Benign |
|  | F | chr4:114214679 CAC/- | c.2472_2474delCAC | p.Thr825del | Likely Benign |
|  | F | chr4:114277716 G/C | c.7942G>C | p.Gly2648Arg | VUS |
|  | M | chr4:114279838A/G | c.10064A>G | p.Gln3355Arg | VUS |
|  | M | chr4:114214588C/T | NM_001148.6: c.2377-8C>T |  | Benign |
|  | F | chr4:114274609C/T | c.4835C>T | p.Thr1612Ile | VUS |
|  | M | chr4:113970924G/A | c.40G>C | p.Glu14Gln | VUS |
|  | F | chr4:114286206 G/A | c.10900G>A | p.Val3634Ile | VUS |
|  | F | chr4:114275950C/T | c.6176C>T | p.Thr2059Met | Likely Benign |
|  | M | chr4:114177035C/T | c.1135C>T | p.Arg379Cys | Likely Benign |
|  | M | chr4:114276826AAGGTC/- | c.7054_7059delGGTCAA | p.Gly2352_Gln2353del | Likely Benign |
|  | M | chr4:114286206 G/A | c.10900G>A | p.Val3634Ile | VUS |
|  | M | chr4:114277889AGA/- | c.8120_8122delAAG | p.Glu2707del | VUS |
|  | F | chr4:114203958C/T | c.2009C>T | p.Ser670Leu | VUS |
|  | M | chr4:114277170C/T | c.7396C>T | p.Arg2466Cys | VUS |
|  | M | chr4:114279563G/T | c.9789G>T | p.Arg3263Ser | VUS |
|  | M | chr4:114284570C/A | c.10833C>A | p.Asp3611Glu | VUS |
|  | F | chr4:114275980G/A | c.6206G>A | p.Arg2069His | Likely Benign |
| ATP13A2 | M | chr1:17322943C/T | c.1244G>A | p.Arg415Gln | VUS |
|  | F | chr1:17332000C/T | c.157G>A | p.Val53Met | VUS |
|  | M | chr1:17313149G/A | c.3314C>T | p.Pro1105Leu | Likely Benign |
|  | F | chr1:17314656T/A | c.2836A>T | p.Ile946Phe | Likely Benign |
|  | F | chr1:17314656T/A | c.2836A>T | p.Ile946Phe | VUS |
|  | M | chr1:17328824C/T | c.602G>A | p.Arg201His | VUS |
|  | M | chr1:17312816C/T | c.3443G>A | p.Arg1148His | VUS |
|  | M | chr1:17330903G/A | c.481C>T | p.Arg161Trp | VUS |
| BDNF | / |  |  |  |  |
| C5orf24 | M | chr5:134190741A/G | c.151A>G | p.Met51Val | VUS |
| CHCHD2 | / |  |  |  |  |
| COMT | F | chr22:19951172 C/T | c.373C>T | p.Arg125Cys | VUS |
|  | M | chr22:19950142 G/A | c.93G>A | p.Trp31* | VUS |
| CTSB | F | chr8:11710930 G/T | c.34C>A | p.Leu12Met | Likely Benign |
|  | M | chr8:11705206 T/C | c.658A>G | p.Lys220Glu | Likely Benign |
|  | M | chr8:11702657C/T | c.997G>aq | p.Asp333Asn | Likely Benign |
| DNAH1 | M | chr3:52394334T/C | c.4579T>C | p.Trp1527Arg | VUS |
|  | F | chr3:52406004A/G | c.6568A>G | p.Met2190Val | Likely Benign |
|  | F | chr3:52418934G/A | c.8455G>A | p.Gly2819Arg | VUS |
|  | M | chr3:52420842C/G | c.8976C>G | p.Phe2992Leu | VUS |
|  | F | chr3:52378663 G/A | c.1444G>A | p.Gly482Arg | VUS |
|  | M | chr3:52380742-/GG | c.1912_1913dupGG | p.Asp638fs*10 | Pathogenic |
|  | F | chr3:52400556A/C | c.5602A>C | p.Lys1868Gln | VUS |
|  | M | chr3:52360334C/T | NM_015512.4:c.581+4C>T |  | VUS |
|  | F | chr3:52404770 G/T | c.6454G>T | p.Asp2152Tyr | VUS |
|  | M | chr3:52394379 A/G | c.4624A>G | p.Ile1542Val | Likely Benign |
|  | M | chr3:52379649T/C | c.1583T>C | p.Leu528Pro | VUS |
|  | M | chr3:52392695G/A | c.4208G>A | p.Arg1403His | VUS |
|  | M | chr3:52426885G/A | c.10318G>A | p.Asp3440Asn | VUS |
|  | M | chr3:52416395G/A | c.7865G>A | p.Arg2622Gln | VUS |
|  | F | chr3:52365180G/C | c.888G>A | p.Gln296His | Likely Benign |
|  | M | chr3:52433148AAC/- | c.12373_12375delACA | p.Thr4125del | VUS |
|  | M | chr3:52428673C/G | c.10819C>G | p.His3607Asp | VUS |
|  | F | chr3:52427469 C/T | c.10594C>T | p.Arg3532Cys | VUS |
|  | M | chr3:52424975 C/G | c.9646C>G | p.Leu3216Val | Likely Benign |
|  | M | chr3:52403889G/A | c.5992G>A | p.Ala1998Thr | VUS |
|  | M | chr3:52419383A/T | c.8545A>T | p.Met2849Leu | VUS |
|  | M | chr3:52404199T/C | c.6212T>C | p.Leu2017Pro | VUS |
|  | M | chr3:52429432G/A | c.11077G>A | p.Gly3693Lys | VUS |
| DNAJC13 | M | chr3:132224222A/C | c.4961A>C | p.Glu1654Ala | VUS |
|  | M | chr3:132203494 C/T | c.3245C>T | p.Thr1082Ile | Likely Benign |
|  | M | chr3:132244562G/T | c.6169G>T | p.Ala2057Ser | Likely Benign |
|  | F | chr3:132203494 C/T | c.3245C>T | p.Thr1082Ile | Likely Benign |
|  | M | chr3:132173004A/AT | NM_015268.3:c.932+16dupT |  | Benign |
|  | M | chr3:132193770G/A | NM_015268.4: c.2292-6G>A |  | Benign |
|  | F | chr3:132218658G/A | NM_015268.4: c.4416+6G>A |  | VUS |
|  | F | chr3:132211341A/G | c.3707A>G | p.Tyr1236Cys | VUS |
|  | M | chr3:132221195 G/C | c.4599G>C | p.Trp1533Cys | VUS |
|  | F | chr3:132169647T/G | c.493T>G | p.Tyr165Asp | VUS |
|  | F | chr3:132217994G/A | c.4181G>A | p.Gly1394Glu | VUS |
|  | F | chr3:132153469ATTC/A | NM_015268.3:c.68+10_68+12delCTT |  | VUS |
| DNAJC6 | M | chr1:65871708 C/T + chr1:65851423 G/A | c.2383C>T + c.829G>A | p.Gln785* + p.Ala277Thr | Likely Pathogenic + VUS |
|  | F | chr1:65864505C/T | c.2048C>T | p.Thr683Met | VUS |
|  | F | chr1:65871598G/A | c.2273G>A | p.Gly758Asp | VUS |
|  | M | chr1:65858354C/T | c.1709C>T | p.Pro570Leu | VUS |
|  | M | chr1:65855060T/C | c.1315T>C | p.Cys439Arg | VUS |
|  | M | chr1:65858186A/G | c.1541A>G | p.Asp514Gly | VUS |
|  | F | chr1:65858132C/T | c.1487C>T | p.Ser496Leu | VUS |
| DRD2 | / |  |  |  |  |
| DRD3 | / |  |  |  |  |
| DYRK1A | M | chr21:38884369-/CAC | c.1815_1817dupCCA | p.His606dup | VUS |
|  | M | chr21:38884668A/G | c.2099G>A | p.Arg700His | VUS |
|  | F | chr21:38878543T/C | c.1688T>C | p.Val563Ala | VUS |
|  | M | chr21:3884713C/G | c.2144C>G | p.Ala715Gly | Likely Benign |
| FBRSL1 | F | chr12:133146665G/A | c.845G>A | p.Arg282His | Likely Benign |
|  | F | chr12:133160162C/T | c.2807C>T | p.Ala936Val | VUS |
|  | M | chr12:133084736C/G | NM_001142641.1:c.292-3C>G |  | VUS |
|  | M | chr12:133067447 | NM_001142641.1:c.291_291+1insAAGGATATGGCCCTGAAGCCACATGAGCGGAAGGAGAA | NP_001136113.1:p.Trp111ArgfsTer5 | VUS |
|  | M | chr12:133153497 G/A | c.1814G>A | p.Arg605Gln | VUS |
|  | M | chr12:133146722C/T | c.902C>T | p.Pro301Leu | Likely Benign |
|  | F | chr12:133084826C/A | c.379C>A | p.Pro127Thr | VUS |
|  | M | chr12:133151100G/A | c.1651G>A | p.Val594Met | VUS |
|  | M | chr12:133146644C/T + chr12:133150995C/T | c.824C>T + c.1675C>T | p.Pro275Leu + p.Arg559Trp | VUS + VUS |
|  | F | chr12:133160080C/T | c.2725C>T | p.His909Tyr | VUS |
|  | M | chr12:133159966G/A | c.2611G>A | p.Ala871Thr | VUS |
|  | M | chr12:133084736C/G | NM_001142641.1:c.292-3C>G |  | VUS |
| FBXO7 | M | chr22:32875089A/G | c.244A>G | p.Ile82Val | Likely Benign |
|  | F | chr22:32894401G/A | c.1453G>A | p.Val485Ile | Likely Benign |
|  | M | chr22:32875119G/C | c.274G>C | p.Asp92His | VUS |
|  | F | chr22:32887070T/C | NM_012179.4: c.872-3T>C |  | VUS |
| FGF20 | / |  |  |  |  |
| FYN | F | chr6:112041068C/T | c.187G>A | p.Val63Ile | VUS |
|  | M | chr6:112020774C/T | c.797G>A | p.Arg266His | VUS |
| GALC | F | chr14:88454869TA/T | NM_000153.3:c.196-3delT |  | VUS |
|  | M | chr14:88454869TA/T | NM_000153.3:c.196-3delT |  | VUS |
|  | M | chr14:88406262 G/A | c.1898C>T | p.Thr633Met | Pathogenic |
|  | M | chr14:88454482T/C | NM_000153.3:c.328+6A>G |  | Likely Benign |
|  | M | chr14:88454869T/TA | NM_000153.3:c.196-3dupT |  | VUS |
|  | M | chr14:88454869TA/T | NM_000153.3:c.196-3delT |  | VUS |
|  | F | chr14:88454869TA/T | NM_000153.3:c.196-3delT |  | VUS |
|  | F | chr14:88454869TA/T | NM_000153.3:c.196-3delT |  | VUS |
|  | M | chr14:88454869TA/T | NM_000153.3:c.196-3delT |  | VUS |
|  | M | chr14:88454869TA/T | NM_000153.3:c.196-3delT |  | VUS |
| GALNT14 | F | chr2:31181310G/A | c.419C>T | p.Thr140Met | VUS |
|  | M | chr2:31215756C/G | c.247G>C | p.Glu83Gln | VUS |
| **GBA** | **M** | **chr1:155206037 G/A** | **c.1223C>T** | **p.Thr408Met** | **Likely Benign** |
|  | **M** | **chr1:155205634T/C** | **c.1226A>G** | **p.Asn409Ser** | **VUS** |
|  | **M** | **chr1:155205518C/G** | **c.1342G>C** | **p.Asp448His (old 409)** | **Pathogenic** |
|  | **M** | **chr1:155207249A/C** | **c.882T>G** | **p.His294Gln (old255)** | **Likely Benign** |
|  | **F** | **chr1:155206052C/G** | **c.1208G>C** | **p.Ser403Thr (old 364)** | **VUS** |
|  | **M** | **chr1:155205518C/G** | **c.1342G>C** | **p.Asp448His (old 409)** | **Pathogenic** |
|  | **F** | **chr1:155206037 G/A** | **c.1223C>T** | **p.Thr408Met** | **Likely Benign** |
|  | **F** | **chr1:155206036 C/T + chr1:155206037 G/A** | **c.1224G>A + c.1223C>T** | **p.Thr408Thr (variante di splicing) + p.Thr408Met** | **Likely Benign + Likely Benign** |
|  | **F** | **chr1:155205043 A/G** | **c.1448T>C** | **p.Leu483Pro (old444)** | **Likely Pathogenic** |
|  | **M** | **chr1:155207245G/A** | **c.886C>T** | **p.Arg296* (old 257)** | **Pathogenic** |
|  | **F** | **chr1:155206037G/A** | **c.1223C>T** | **p.Thr408Met (old369)** | **Benign** |
|  | **F** | **chr1:155205634T/C** | **c.1226A>G** | **p.Asn409Ser (old 370)** | **Likely Pathogenic** |
|  | **M** | **chr1:155205043 A/G** | **c.1448T>C** | **p.Leu483Pro (old444)** | **Likely Pathogenic** |
|  | **M** | **chr1:155208087A/T** | **c.599T>A** | **p.Ile200Asn** | **VUS** |
|  | **F** | **chr1:155208420C/T** | **c.476G>A** | **p.Arg159Gln (old 119)** | **VUS** |
|  | **M** | **chr1:155208414 G/A** | **c.482C>T** | **p.Pro161Leu** | **Likely Benign** |
| GBF1 | F | chr10:104117868 C/T | c.712C>T | p.Arg238Cys | Likely Benign |
|  | M | chr10:104135150 A/G | c.3692A>G | p.Lys1231Arg | VUS |
|  | M | chr10:104136187 G/A | c.4042G>A | p.Val1348Met | Likely Benign |
|  | M | chr10:104123030 G/A | c.1891G>A | p.Asp631Asn | VUS |
|  | M | chr10:104117923C/G | c.767C>G | p.Thr256Ser | VUS |
|  | F | chr10:104129629C/T | c.3224C>T | p.Thr1076Ile | VUS |
|  | M | chr10:104117868 C/T | c.712C>T | p.Arg238Cys | Likely Benign |
|  | M | chr10:104135131C/T | c.3673C>T | p.Arg1225Cys | VUS |
|  | F | chr10:104139308 G/A | c.4661G>A | p.Arg1554His | VUS |
|  | M | chr10:104139298G/A | c.4651G>A | p.Asp1551Asn | VUS |
|  | F | chr10:104140043C/G | c.4901C>G | p.Ser1634Cys | VUS |
|  | M | chr10:104140897G/A | c.5172G>A | p.Met1724Ile | VUS |
| GCH1 | M | chr14:55369356G/A | c.26C>T | p.Pro9Leu | VUS |
|  | F | chr14:55310817T/C | c.671A>G | p.Lys224Arg | VUS |
| GRIN2A | M | chr16:9857012 A/C | c.4389T>G | p.Asp1463Glu | VUS |
|  | M | chr16:10032064A/G | c.760T>C | p.Phe254Leu | VUS |
|  | M | chr16:10032404G/A + chr16:9858211T/C | c.419C>T + c.3191A>G | p.Pro140Leu + p.Thr1064Ala | VUS + Likely bening |
|  | M | chr16:9857094T/C | c.4307A>G | p.Asn1436Ser | Likely Benign |
|  | F | chr16:9934825C/T | c.1465G>A | p.Val489Ile | VUS |
|  | M | chr16:9857823A/C | c.3578T>G | p.Leu1193Trp | VUS |
| ITPKB | M | chr1:226924280C/A | c.880G>T | p.Gly294Trp | VUS |
|  | F | chr1:226822473C/T | c.2740G>A | p.Val914Ile | Likely Benign |
|  | M | chr1:226925103C/G | c.57G>C | p.Glu19Asp | VUS |
|  | M | chr1:226924880 C/G | c.280G>C | p.Gly94Arg | VUS |
|  | M | chr1:226924735 T/C | c.425A>G | p.Gln142Arg | VUS |
|  | M | chr1:226923391 C/T | c.1769G>A | p.Arg590Gln | VUS |
|  | M | chr1:226923989T/C | c.1171A>G | p.Lys391Glu | Likely Benign |
|  | M | chr1:226827322G/T | c.2489C>A | p.Thr830Asn | VUS |
| LAMP2 | F | chrX:119582966C/T | c.415G>A | p.Glu139Lys | Likely Benign |
| LRRK2 | M | chr12:40699666A/G | c.3857A>G | p.Asn1286Ser | VUS |
|  | F | chr12:40619447A/C | NM_198578.3:c.237+5A>C |  | VUS |
|  | F | chr12:40687426G/C | c.2769G>C | p.Gln923His | Likely Benign |
|  | F | chr12:40734202G/A | c.6055G>A | p.Gly2019Ser | Likely Benign |
|  | M | chr12:40631875T/C | c.541T>C | p.Cys181Arg | VUS |
|  | F | chr12:40688648-40688716del | … |  | VUS |
|  | M | chr12:40787428G/A | c.2771G>A | p.Arg924His | Likely Benign |
|  | M | chr12:40734202G/A | c.6055G>A | p.Gly2019Ser | Likely Benign |
|  | M | chr12:40716270 C/A | c.5467C>A | p.Gln1823lys | Likely Benign |
|  | M | chr12:40704236C/T | c.4321C>T | p.Arg1441Cys | Pathogenic |
|  | M | chr12:40734202G/A | c.6055G>A | p.Gly2019Ser | Likely Benign |
|  | F | chr12:40697875A/C | c.3716A>C | p.Glu1239Ala | VUS |
|  | F | chr12:40643624T/G | NM_198578.3:c.839-4T>G |  | VUS |
|  | M | chr12:40704236C/T | c.4321C>T | p.Arg1441Cys | Pathogenic |
|  | M | chr12:40702474G/A | c.4165G>A | p.Val1389Ile | Likely Benign |
|  | F | chr12:40619009G/A | c.76G>A | p.Val26Ile | VUS |
|  | F | chr12:40757242 C/T | c.7067C>T | p.Thr2356Ile | Likely Benign |
| MAD2L2 | M | chr1:11736965 T/C | c.272A>G | p.Glu91Gly | VUS |
| MAOB | F | chrX:43655091T/C | c.663A>G | p.Ile221Met | VUS |
| MAP7 | M | chr6:136687478G/A | c.1066C>T | p.Arg356Cys | Likely Benign |
|  | M | chr6:136693745A/T | c.860T>A | p.Ile287Asn | Likely Benign |
|  | M | chr6:136693665G/T | c.940C>A | p.Arg314Ser | VUS |
|  | F | chr6:136742876A/C | c.195T>G | p.Ile65Met | VUS |
|  | M | chr6:136871500C/T | c.47G>A | p.Gly16Asp | VUS |
|  | F | chr6:136698944 G/A | c.766C>T | p.His256Tyr | VUS |
|  | M | chr6:136687459A/G | NM_001198609.1:c.1079+6T>C |  | Likely Benign |
|  | M | chr6:136709644C/T | c.479G>A | p.Arg160His | VUS |
|  | M | chr6:136687478G/A | c.1066C>T | p.Arg356Cys | Likely Benign |
| MAPT | M | chr17:44061015 C/T | c.1070C>T | p.Ser357Leu | Likely Benign |
|  | M | chr17:44067365C/T | c.1529C>T | p.Pro510Leu | VUS |
| MBNL2 | M | chr13:98009847 A/G | c.970A>G | p.Thr324Ala | VUS |
|  | M | chr13:97999297-/GCCGCG | c.790_795dupGCGGCC | p.Ala264_Ala265dup | VUS |
| NIBAN2 | F | chr9:130269273G/C | c.2092C>G | p.Leu698Val | VUS |
|  | F | chr9:130269273G/C | c.2092C>G | p.Leu698Val | VUS |
|  | M | chr9:130269676G/T + chr9:130279256C/T | c.1689C>A + c.853G>A | p.Asn563Lys + p.Ala285Thr | VUS + VUS |
|  | M | chr9:130287331C/A | NM_022833.2:c.421+6G>T |  | VUS |
|  | F | chr9:130269527C/T | c.1838C>T | p.Ser613Leu | VUS |
| NOD2 | M | chr16:50745137C/T | c.1234C>T | p.Arg412Cys | Likely Benign |
|  | M | chr16:50750505G/A | c.2389G>A | p.Asp797Asn | Likely Benign |
|  | F | chr16:50744753 C/T | c.850C>T | p.Arg284Trp | Likely Benign |
|  | M | chr16:50733392T/A | NM_022162.1:c.74-7T>A |  | Benign |
|  | F | chr16:50745201A/G | c.1298A>G | p.His433Arg | VUS |
|  | F | chr16:50753834A/G | NM_022162.1:c.2631-2A>G |  | VUS |
|  | F | chr16:50733392T/A | NM_022162.1:c.74-7T>A |  | Benign |
|  | F | chr16:50745099G/A | c.1196G>A | p.Arg399His | VUS |
|  | M | chr16:50744753 C/T | c.850C>T | p.Arg284Trp | Likely Benign |
|  | M | chr16:50744753 C/T | c.850C>T | p.Arg284Trp | Likely Benign |
|  | M | chr16:50750582G/GT | NM_022162.1:c.2546+2dupT |  | VUS |
|  | M | chr16:50746199G/A | c.2296G>A | p.Val793Met | Likely Benign |
|  | F | chr16:50744565T/G | c.662T>G | p.Leu221Arg | Likely Benign |
|  | M | chr16:50750505G/A | c.2389G>A | p.Asp797Asn | Likely Benign |
|  | M | chr16:50750562G/A | c.2446G>A | p.Glu816Lys | VUS |
|  | F | chr16:50744999C/T | c.1096C>T | p.Arg366Cys | VUS |
| OPRK1 | M | chr8:54163541 G/T | c.57C>A | p.Ser19Arg | VUS |
|  | M | chr8:54163541 G/T | c.57C>A | p.Ser19Arg | VUS |
|  | F | chr8:54163541 G/T | c.57C>A | p.Ser19Arg | VUS |
| OPRM1 | M | chr6:154412516 C/T | c.1073C>T | p.Ser358Phe | VUS |
|  | F | chr6:154567884C/A | c.1222C>A | p.Arg408Ser | VUS |
|  | M | chr6:154412516 C/T | c.1073C>T | p.Ser358Phe | VUS |
|  | F | chr6:154414561G/A | c.1321G>A | p.Gly441Arg | VUS |
|  | F | chr6:154439814T/C | NM_001145279.2:c.1444-4T>C |  | VUS |
|  | F | chr6:154412194 A/G | c.751A>G | p.Thr251Ala | VUS |
|  | M | chr6:154411134C/T | c.464C>T | p.Thr155Ile | VUS |
| PARK7 | M | chr1:8037768C/T | c.379C>T | p.Pro127Ser | VUS |
|  | M | chr1:8045079G/A | c.535G>A | p.Ala179Thr | Likely Benign |
|  | F | chr1:8030993C/T | c.292C>T | p.Arg98Trp | VUS |
|  | M | chr1:8029466T/TA | NM_007262.4:c.252+8dupA |  | VUS |
| PINK1 | / |  |  |  |  |
| PRKN | M | chr6:162394349G/A | c.719C>T + del. esone 3 | p.Thr240Met | Likely Pathogenic |
|  | F | chr6:162683764T/C | c.205A>G | p.Ile69Val | Likely Benign |
|  | F | chr6:162864433T/G | c.80A>C | p.Lys27Thr | VUS |
|  | **F** | **chr6:162864411CT/-** | **c.101_102delAG + dupl., esone 3** | **p.Gln34Argfs*5** | **Pathogenic** |
| PTRHD1 | / |  |  |  |  |
| RAB39B | / |  |  |  |  |
| **RABGEF1** | / |  |  |  |  |
| SH3GL2 | F | chr9:17789543A/G | c.619A>G | p.Met207Val | VUS |
| SIPA1L2 | F | chr1:232649746C/G | c.1340G>C | p.Ser447Thr | VUS |
|  | F | chr1:232649746C/G | c.1340G>C | p.Ser447Thr | VUS |
|  | M | chr1:232581473G/A | c.3155C>T | p.Thr1052Ile | VUS |
|  | M | chr1:232575219A/T + chr1:232596682T/C | c.3666T>A + c.3046A>G | p.Ser1222Arg + p.Thr1016Ala | VUS + VUS |
|  | M | chr1:232650410 C/T | c.676G>A | p.Val226Ile | VUS |
|  | M | chr1:232629409A/G | NM_020808.3:c.1484-3T>C |  | VUS |
|  | M | chr1:232538135T/C | NM_020808.3:c.5022+3A>G |  | VUS |
|  | M | chr5:134193062 C/G | c.443C>G | p.Ala148Gly | VUS |
|  | F | chr1:232574927C/T | c.3958G>A | p.Ala1320Thr | VUS |
| SLC6A3 | F | chr5:1432818C/A | NM_001044.4:c.419-5G>T | 2,81*10--5 | Likely Benign |
| SNCA | / |  |  |  |  |
| STAB1 | F | chr3:52554966CAC/- | c.5860_5862delACC | p.Thr1954del | VUS |
|  | F |  | c.5611_5644del34 | p.Ala1871Aspfs*2 | VUS |
|  | F | chr3:52551020G/C | c.4384G>C | p.Gly1462Arg | VUS |
|  | M | chr3:52546624 A/G | c.2992A>G | p.Asn998Asp | VUS |
|  | M | chr3:52537368C/T | c.703C>T | p.Pro235Ser | VUS |
|  | F | chr3:52539696 C/A | c.1594C>A | p.Pro532Thr | VUS |
|  | F | chr3:52550239C/A | c.4129C>A | p.Pro1377Thr | VUS |
|  | M | chr3:52536185 G/A | c.428G>A | p.Arg143His | VUS |
|  | F | chr3:52556690 T/C | c.6730T>C | p.Ser2244Pro | VUS |
|  | M | chr3:52536185 G/A | c.428G>A | p.Arg143His | VUS |
|  | F | chr3:52555413G/A | c.5945G>A | p.Arg1982His | VUS |
|  | F | chr3:52537450A/C | c.785A>C | p.Asn262Thr | VUS |
|  | F | chr3:52556184C/G | c.6403C>G | p.Pro2135Ala | Benign |
|  | F | chr3:52548787C/T | c.3749C>T | p.Ser1250Leu | VUS |
|  | M | chr3:52546403C/A | c.2930C>A | p.Pro977His | Likely Benign |
|  | M | chr3:52547907-/C | c.3364dupC | p.Arg1122Profs*37 | VUS |
|  | F | chr3:52555698C/T | c.6145C>T | p.Arg2049Cys | VUS |
|  | F | chr3:52546624 A/G | c.2992A>G | p.Asn998Asp | VUS |
|  | F | chr3:52557775G/A | NM_015136.2:c.7395+3G>A |  | VUS |
|  | M | chr3:52535713 G/A | c.275G>A | p.Arg92Gln | VUS |
|  | M | chr3:52550455 C/T | c.4228C>T | p.Arg1410Cys | VUS |
|  | M | chr3:52539019A/T | c.1378A>T | p.Lys460* | VUS |
|  | M | chr3:52550712G/A | c.4291G>A | p.Val1431Met | Likely Benign |
|  | F | chr3:52550470G/C | NM_015136.2:c.4240+3G>C |  | VUS |
| SV2C | M | chr5:75596655T/C | c.1738T>C | p.Tyr580His | VUS |
| SYNJ1 | F | chr21:34050957C/T | c.1508G>A | p.Arg503His | VUS |
|  | F | chr21:34100290_34100301 del CAGCCGCCGCCA | c.51_62delTGGCGGCGGCTG | p.Gly18_Cys21del | VUS |
|  | M | chr21:34099166 T/C | c.41A>G | p.Asp14Gly | VUS |
|  | F | chr21:34011387G/A | c.3746C>T | p.Pro1249Leu | Likely Benign |
| TMEM175 | M | chr4:947004C/G | c.489C>G | p.His163Gln | VUS |
|  | M | chr4:945477T/C | c.2T>C | p.Met1? | VUS |
|  | M | chr4:951893G/A | c.1124G>A | p.Arg375His | VUS |
|  | F | chr4:951622G/A | c.853G>A | p.Val285Ile | VUS |
| TMEM230 | M | chr20:5093674 T/C | c.1A>G | p.Met1? | VUS |
|  | F | chr20:5090075A/G | c.191T>C | p.Met64Thr | VUS |
|  | M | chr20:5093674 T/C | c.1A>G | p.Met1? | VUS |
| VAMP4 | / |  |  |  |  |
| VPS13C | M | chr15:62172801A/G | NM_020821.2:c.10002+7T>C |  | VUS |
|  | F | chr15:62270938T/C | c.2177A>G | p.Lys726arg | VUS |
|  | F | chr15.62246761G/T | NM_020821.2:c.4166-8C>A |  | Benign |
|  | F | chr15:62246733C/T | c.4186G>A | p.Glu1396Lys | VUS |
|  | F | chr15:62148583C/G | c.10978G>C | p.Glu3660Gln | VUS |
|  | **M** | **chr15:62146733C/T + chr15:62212349T/A** | **c.11185G>A + c.7394A>T** | **p.Ala3729Thr + p.Glu2465Val** | **VUS + VUS** |
|  | M | chr15:62182533 G/A | c.9172C>T | p.Arg3058Cys | VUS |
|  | F | chr15:62273685C/T | NM_020821.2:c.2030-8G>A |  | VUS |
|  | M | chr15:62207966G/T | c.8311C>A | p.Pro2771Thr | VUS |
|  | F | chr15:62147087C/A | c.11143G>T | p.Asp3715Tyr | Likely Benign |
|  | F | chr15:62209608T/C | c.7987A>G | p.Ile2663Val | VUS |
|  | F | chr15:62246761G/T | NM_020821.2:c.4166-8C>A |  | Benign |
|  | F | chr15:62244082T/C | c.4397A>G | p.Tyr1466Cys | VUS |
|  | M | chr15:62170873G/A | c.10075C>T | p.His3359Tyr | VUS |
|  | M | chr15:62214756 A/G | c.6815T>C | p.Ile2272Thr | Likely Benign |
|  | M | chr15:62167155CA/C | NM_020821.2:c.10339-6delT |  | VUS |
|  | M | chr15:62254707G/A | c.3466C>T | p.Arg1156Cys | VUS |
|  | M | chr15:62167155CA/C | NM_020821.2:c.10339-6delT |  | VUS |
|  | F | chr15:62172897C/T | c.9913G>A | p.Asp3305Asn | VUS |
|  | M | chr15:62204098C/T | c.8656G>A | p.Glu2882Lys | VUS |
|  | M | chr15:62182367G/T | c.9338C>A | p.Thr3113Asn | VUS |
| VPS35 | F | chr16:46715983A/G | NM_018206.4:c.199+8T>C | 1,03*10-3+6omo | Benign |
|  | M | chr16:46708574TAA/T | NM_018206.4:c.915-4_915-3delTT |  | Likely Benign |
| **bold: Pathogenic variants** | | | | | |

Abbreviations: HGVSc: Human Genome Variations Society for a coding DNA reference sequence; HGVSp: Human Genome Variations Society for a protein reference sequence; ACGM: American College of Medical Genetics and Genomics; eVai: Expert Variant Interpreter.
