# Supplementary material for: Gender Is the Main Predictor of Wearing‐Off and Dyskinesia in Levodopa‐Naïve Patients with Parkinson's Disease
Source: Mov Disord Clin Pract. 2025 May 29;12(11):1774–83. doi: 10.1002/mdc3.70143 (PMC12625146; doi:10.1002/mdc3.70143)
Supplement: Supplementary file 5 — Table S4. Absolute and percentage frequencies of patients with at least one adverse event throughout the study by gender and overall—all included patients. [file MDC3-12-1774-s001.docx]

###### Supplementary Table 4: Absolute and percent frequencies of patients with at least one Adverse Event throughout the study,

###### by gender and overall – All included patients

__________________________________________________________________________________________________________________________________

Male Female ALL

Statistics (N=174) (N=115) (N=289)

_________________________________________________________________________________________________________________________________

Number of patients with at least one AE n (%) 25 (14.4%) 25 (21.7%) 50 (17.3%)

Cardiac disorders

Palpitations n (%) 1 (50.0%) 1 (20.0%) 2 (28.6%)

Arrhythmia n (%) 1 (50.0%) 0 (0.0%) 1 (14.3%)

Cardiac arrest n (%) 0 (0.0%) 1 (20.0%) 1 (14.3%)

Chest pain n (%) 0 (0.0%) 1 (20.0%) 1 (14.3%)

Extrasystoles n (%) 0 (0.0%) 1 (20.0%) 1 (14.3%)

Tachycardia n (%) 0 (0.0%) 1 (20.0%) 1 (14.3%)

Ear and labyrinth disorders

Tinnitus n (%) 0 (0.0%) 1 (100.0%) 1 (100.0%)

Endocrine disorders

Goitre n (%) 0 (0.0%) 1 (100.0%) 1 (100.0%)

Gastrointestinal disorders

Nausea n (%) 1 (33.3%) 2 (33.3%) 3 (33.3%)

Abdominal pain n (%) 0 (0.0%) 1 (16.7%) 1 (11.1%)

Dyspepsia n (%) 0 (0.0%) 1 (16.7%) 1 (11.1%)

Gastritis n (%) 0 (0.0%) 1 (16.7%) 1 (11.1%)

Gastrooesophageal reflux disease n (%) 1 (33.3%) 0 (0.0%) 1 (11.1%)

Inguinal hernia n (%) 1 (33.3%) 0 (0.0%) 1 (11.1%)

Tooth disorder n (%) 0 (0.0%) 1 (16.7%) 1 (11.1%)

General disorders and administration site conditions

Oedema peripheral n (%) 1 (33.3%) 1 (50.0%) 2 (40.0%)

Asthenia n (%) 0 (0.0%) 1 (50.0%) 1 (20.0%)

Hepatobiliary disorders

Hepatic steatosis n (%) 0 (0.0%) 1 (100.0%) 1 (100.0%)

Infections and infestations

COVID-19 n (%) 0 (0.0%) 4 (100.0%) 4 (80.0%)

COVID-19 pneumonia n (%) 1 (100.0%) 0 (0.0%) 1 (20.0%)

Injury, poisoning and procedural complications

Fall n (%) 1 (100.0%) 3 (100.0%) 4 (100.0%)

Investigations

Hepatic enzyme increased n (%) 0 (0.0%) 1 (100.0%) 1 (100.0%)

Metabolism and nutrition disorders

Vitamin D deficiency n (%) 0 (0.0%) 2 (50.0%) 2 (50.0%)

Increased appetite n (%) 0 (0.0%) 1 (25.0%) 1 (25.0%)

Type 2 diabetes mellitus n (%) 0 (0.0%) 1 (25.0%) 1 (25.0%)

Musculoskeletal and connective tissue disorders

Back pain n (%) 0 (0.0%) 2 (50.0%) 2 (50.0%)

Lumbar spinal stenosis n (%) 0 (0.0%) 1 (25.0%) 1 (25.0%)

Osteoporosis n (%) 0 (0.0%) 1 (25.0%) 1 (25.0%)

Neoplasms benign, malignant and unspecified (incl

cysts and polyps)

Adenocarcinoma of prostate n (%) 1 (50.0%) 0 (0.0%) 1 (50.0%)

Metastatic neoplasm n (%) 1 (50.0%) 0 (0.0%) 1 (50.0%)

Nervous system disorders

Cognitive disorder n (%) 2 (40.0%) 0 (0.0%) 2 (28.6%)

Cerebrovascular disorder n (%) 0 (0.0%) 1 (50.0%) 1 (14.3%)

Dyskinesia n (%) 1 (20.0%) 0 (0.0%) 1 (14.3%)

Hypoaesthesia n (%) 1 (20.0%) 0 (0.0%) 1 (14.3%)

Neuralgia n (%) 0 (0.0%) 1 (50.0%) 1 (14.3%)

Transient ischaemic attack n (%) 1 (20.0%) 0 (0.0%) 1 (14.3%)

Psychiatric disorders

Rapid eye movement sleep behaviour disorder n (%) 2 (18.2%) 3 (42.9%) 5 (27.8%)

Depressed mood n (%) 2 (18.2%) 1 (14.3%) 3 (16.7%)

Depression n (%) 2 (18.2%) 1 (14.3%) 3 (16.7%)

Mixed anxiety and depressive disorder n (%) 2 (18.2%) 0 (0.0%) 2 (11.1%)

Anxiety n (%) 1 (9.1%) 0 (0.0%) 1 (5.6%)

Hallucination n (%) 0 (0.0%) 1 (14.3%) 1 (5.6%)

Insomnia n (%) 1 (9.1%) 0 (0.0%) 1 (5.6%)

Psychotic disorder n (%) 0 (0.0%) 1 (14.3%) 1 (5.6%)

Sleep disorder n (%) 1 (9.1%) 0 (0.0%) 1 (5.6%)

Reproductive system and breast disorders

Benign prostatic hyperplasia n (%) 2 (100.0%) 0 (0.0%) 2 (100.0%)

Respiratory, thoracic and mediastinal disorders

Cough n (%) 0 (0.0%) 2 (100.0%) 2 (100.0%)

Skin and subcutaneous tissue disorders

Rash erythematous n (%) 0 (0.0%) 1 (100.0%) 1 (100.0%)

Surgical and medical procedures

Cardiac pacemaker insertion n (%) 1 (100.0%) 0 (0.0%) 1 (100.0%)

Vascular disorders

Deep vein thrombosis n (%) 0 (0.0%) 2 (50.0%) 2 (50.0%)

Orthostatic hypotension n (%) 0 (0.0%) 1 (25.0%) 1 (25.0%)

Phlebitis n (%) 0 (0.0%) 1 (25.0%) 1 (25.0%)
