# Supplementary material for: Gender Is the Main Predictor of Wearing‐Off and Dyskinesia in Levodopa‐Naïve Patients with Parkinson's Disease
Source: Mov Disord Clin Pract. 2025 May 29;12(11):1774–83. doi: 10.1002/mdc3.70143 (PMC12625146; doi:10.1002/mdc3.70143)
Supplement: Supplementary file 6 — Table S5. Absolute and percentage frequencies of patients with at least one serious adverse event throughout the study by gender and overall—all included patients. [file MDC3-12-1774-s006.docx]

**Supplementary Table 5: Absolute and percent frequencies of patients with at least one serious adverse event**

**throughout the study, by gender and overall - All included patients**

___________________________________________________________________________________________________________________

Male Female ALL

Statistics (N=174) (N=115) (N=289)

n (%) n (%) n (%)

__________________________________________________________________________________________________________________

Number of patients with at least one SAE 7 (4.0%) 2 (1.7%) 9 (3.1%)

Cardiac disorders

Cardiac arrest (causing death) 0 (0.0%) 1 (100.0%) 1 (100.0%)

Gastrointestinal disorders

Inguinal hernia 1 (100.0%) 0 (0.0%) 1 (100.0%)

General disorders and administration site conditions

Death (undefined cause) 1 (100.0%) 0 (0%) 1 (100.0%)

Infections and infestations

COVID-19 pneumonia (causing death) 1 (100.0%) 0 (0.0%) 1 (100.0%)

Neoplasms benign, malignant and unspecified (incl

cysts and polyps)

Adenocarcinoma of prostate 1 (50.0%) 0 (0.0%) 1 (50.0%)

Metastatic neoplasm (causing death) 1 (50.0%) 0 (0.0%) 1 (50.0%)

Nervous system disorders

Transient ischaemic attack 1 (100.0%) 0 (0.0%) 1 (100.0%)

Surgical and medical procedures

Cardiac pacemaker insertion 1 (100.0%) 0 (0.0%) 1 (100.0%)

Vascular disorders

Deep vein thrombosis 0 (0.0%) 1 (100.0%) 1 (100.0%)

_____________________________________________________________________________________________________________________
